# Proteomic profiling identifies a stromal TGF-β1/podoplanin axis as a driver of colorectal cancer progression

**DOI:** 10.1186/s13046-025-03496-3

**Published:** 2025-08-22

**Authors:** Silvia Di Agostino, Davide La Padula, Vittoria Rago, Caterina Gabriele, Francesco Conforti, Elio Aprigliano, Lidia Urlandini, Elvira Parrotta, Danilo Lofaro, Francesca Vescio, Andrea Sacconi, Valeria Cernaro, Giuseppe Currò, Angela Alibrandi, Girolamo Ranieri, Valeria Zuccalà, Antonio Ieni, Marco Gaspari, Giovanni Cuda, Michele Ammendola, Vittorio Abbonante

**Affiliations:** 1 Department of Health Sciences, Magna Graecia University of Catanzaro, Catanzaro, 88100 Italy; 2 Department of Pharmacy, Health and Nutritional Sciences, University of Calabria, Rende, 87036 Italy; 3 Department of Experimental and Clinical Medicine, Magna Graecia University of Catanzaro, Catanzaro, 88100 Italy; 4 Pathology Unit, Annunziata Hospital, Cosenza, 87100 Italy; 5 Department of Medical and Surgical Sciences, Magna Graecia University of Catanzaro, Catanzaro, 88100 Italy; 6 Department of Mechanical, Energy, Management Engineering, University of Calabria, Rende, 87036 Italy; 7 Clinical Trial Center, Biostatistics and Bioinformatics, IRCCS Regina Elena National Cancer Institute, Rome, 00144 Italy; 8 Department of Clinical and Experimental Medicine, Unit of Nephrology and Dialysis, A.O.U. “G. Martino”, University of Messina, Messina, 98125 Italy; 9 Department of Human Pathology in Adult and Developmental Age “Gaetano Barresi”, University of Messina, Messina, 98125 Italy; 10 Oncology Unit, IRCCS National Cancer Istitute “Giovanni Paolo II”, Bari, 70100 Italy

**Keywords:** Colorectal cancer, Tumor microenvironment, Proteomic, Tumor associated macrophages, Cancer associated fibroblasts, Transforming growth factor beta, YAP/TAZ

## Abstract

**Background:**

The tumor microenvironment (TME) plays a pivotal role in the development and progression of colorectal cancer (CRC), yet the complex crosstalk among its components remains incompletely understood. Cancer-associated fibroblasts (CAFs) and tumor-associated macrophages (TAMs) have emerged as key regulators of CRC progression, but their specific contributions, particularly given their heterogeneity, are not fully elucidated. This study identifies podoplanin (PDPN), a transmembrane glycoprotein enriched in CAFs, as highly expressed in the CRC TME, in particular surrounding the tumor, and associated with macrophage infiltration and cancer progression.

**Methods:**

We performed mass spectrometry-based proteomic analysis on matched CRC and adjacent normal tissues from patients to identify altered signaling pathways and protein expression. The clinical relevance of PDPN expression was evaluated in CRC samples from two independent cohorts using immunohistochemistry and immunofluorescence analysis. Publicly available data from the Gene Expression Omnibus (GEO) database were analyzed to assess the association between PDPN expression and patient survival. Functional assays using direct and indirect co-culture systems investigated the influence of macrophage infiltration on stromal PDPN expression and its effect on colon adenocarcinoma cell growth.

**Results:**

PDPN expression was significantly elevated in the stroma of the colorectal tumor tissues compared to normal tissues and correlated with M2-like macrophage infiltration. High PDPN expression was associated with reduced relapse-free survival in CRC patients. Stromal cells pre-conditioned with M2-like macrophages upregulated PDPN and more effectively supported the growth of three colon adenocarcinoma cell lines. PDPN depletion impaired the ability of stromal cells to promote tumor cell proliferation. Mechanistically, M2-like macrophage pre-conditioning induced a TGF-β1–dependent increase in YAP/TAZ nuclear localization, RhoA/ROCK/myosin-driven cytoskeletal contractility, and extracellular matrix (ECM) production in stromal cells. Inhibition of TGF-β1 signaling or ROCK activity reduced stromal support for cancer cell growth.

**Conclusion:**

This study reveals a novel mechanism by which the TME facilitates CRC progression and highlights PDPN as a potential prognostic biomarker and therapeutic target in CRC.

**Graphical abstract:**

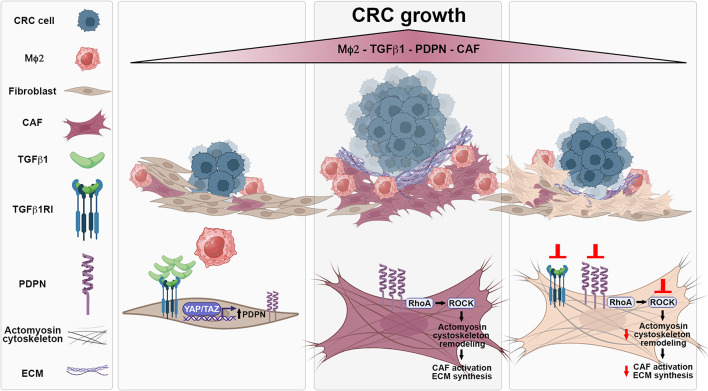

**Supplementary Information:**

The online version contains supplementary material available at 10.1186/s13046-025-03496-3.

## Background

Despite recent advances in the treatment of colorectal cancer (CRC), which is now the third most common malignant neoplasia and the fourth leading cause of death worldwide, the prognosis for patients remains poor [1, 2]. CRC arises from the epithelial mucosa and its progression involves multiple steps and cumulative effects of different factors [3–5]. Over 70% of CRCs are sporadic and associated with mutations in APC, TP53, KRAS, SMAD4 and PIK3CA, 20% have an associated hereditary component, and less than 5% are inherited. However, due to high CRC heterogeneity, very poor genotype-phenotype correlation has emerged [6]. Regarding the molecular stratification of CRC, the Consensus Molecular Subtypes (CMS) Consortium, by analyzing CRC expression profile data from multiple studies, described four CMS groups [6]. CMS4 tumors, characterized by high cancer associated fibroblast (CAF) and macrophage infiltration and lack of T- and NK-cell-mediated response, high epithelial-mesenchymal transition (EMT), TGF-β-associated signatures, and extracellular matrix (ECM) remodeling, have the worst prognosis and a poor response to anti-EGFR drugs and first-line chemotherapy regimens.

The CRC tumor microenvironment (TME) has been reported to be a crucial supporter of tumorigenesis and tumor progression and a determining factor in the therapeutic response [7, 8]. The TME is a complex and heterogeneous ecosystem composed of tumor cells and resident and circulating cells of the intestinal mucosa and submucosa (e.g., stromal cells, adaptive and innate immune cells and endothelial cells). This microenvironment, which is modulated by the CRC to favor its progression and invasion, also includes a dynamic ECM consisting of collagen fibers, elastic fibers, proteoglycans and glycoproteins [8]. Recently, it is becoming increasingly clear that the HIPPO pathway, a highly conserved signaling pathway crucial for the tissue homeostasis, and regeneration, can severely influence the composition of the TME by affecting the activity of CAF and of immune cells [9, 10]. The function of downstream transcriptional cofactors yes-associated protein (YAP1) and transcriptional co-activator with PDZ-binding motif (TAZ/WWTR1), regulates the expression of EMT-related transcription molecules, thus HIPPO pathway deregulation might induce changes that modify the physiological function of the niche pushing it towards a tumor microenvironment phenotype [11, 12].

During the last decade, tumor-associated macrophages (TAMs) have emerged among the most interesting immune cells in the CRC TME [13, 14]. By secreting cytokines and chemokines and coordinating with inflammatory signals from other immune cells, TAMs reprogram TME actors, including fibroblast and mesenchymal cells, thus influencing TME composition and determining tumor progression, metastasis, immune tolerance and drug resistance [13, 15–17]. Through differentiation mechanisms also induced by the ECM, TAMs can exhibit either a classically activated M1 (proinflammatory) phenotype that is able to recognize and kill tumor cells and an alternatively activated M2 (anti-inflammatory) phenotype which, on the contrary, favors a state of immunosuppression around the tumor, promoting its progression [18–20].

Within the TME, CAFs are considered to be the most abundant stromal cells. They promote malignant progression by secreting cytokines, growth factors, and ECM proteins that support tumor cell proliferation, migrations and invasion [21]. Among the emerging markers of CAFs in a variety of malignancies, podoplanin (PDPN) is gaining particular attention [22–25]. In most cancers, high PDPN expression in CAFs is associated with poor patient outcomes, including lymph node metastasis and reduced overall survival. PDPN is a small cell surface glycoprotein playing various and fundamental roles in lymphatic system, heart and alveoli development [26]. Its biological functions which span from the regulation of cell proliferation, contractility and migration to EMT regulation, and remodeling of the ECM are achieved by its interaction to other proteins in the same cell or in adjacent cells. In particular, the interaction of PDPN cytoplasmic tail appeared to be critical in the rearrangement of the actin cytoskeleton and modulation of small Rho GTPases both involved in EMT during cancer [27].

In the present study, we employed a label-free LC-MS/MS-based comparative proteomics, to compare the protein expression profiles in matched normal and CRC tissues. We found that PDPN is upregulated in stromal cells surrounding CRC and validated its potential contribution to colorectal adenocarcinoma cell growth.

## Methods and materials

The antibodies and primers used in this study are listed in Supplementary Tables 1 and 2.

### Patient sample collection

Resection samples from 20 patients with CRC were collected at Azienda Ospedaliero-Universitaria Renato Dulbecco. All materials were collected after prior written informed consent, and the study was approved by the Territorial Ethics Committee for Azienda Ospedaliero-Universitaria Renato Dulbecco, Register Protocol No. 85 of November 28th 2023, Calabria Region. Fresh tissue samples were rapidly processed on ice and subjected to routine pathologic assessment for preparation of formalin-fixed, paraffin-embedded (FFPE) specimens. From tumor samples, necrotic regions were removed, and tissues were cut with scalpels into pieces (∼1–2 mm diameter). In parallel, fresh tissue samples from tumor-distant and tumor-adjacent normal regions were processed. From each sample, three to five randomly chosen pieces were immediately frozen at − 80 °C for subsequent protein isolation, while three to five randomly chosen pieces were immersed in RNAlater (Thermo Fisher Scientific, AM7020) for subsequent RNA isolation. For protein extraction, tissues were homogenized in lysis buffer (4% SDS, 6 M urea, 50 mM TRIS HCl pH8, DTT 10 mM) using GentleMACS tissue dissociator (Miltenyi, 130-093-235) and M tubes (Miltenyi, 130-093-236) and then centrifuged at 15,700 × g at 4 °C for 15 min. RNA extraction was performed by miRNeasy mini kit (Qiagen, 217004) following manufacturer instruction.

A separate independent CRC patient sample cohort of 20 cases was obtained from the archives of the Department of Human Pathology of Adult and Evolutive Age (University of Messina, Messina, Italy). This retrospective analysis has been performed taking into consideration the Good Clinical Practice guidelines as well as the Declaration of Helsinki (revised in 2013). No informed consent was required since the nature of the study is retrospective; however, at the admission in the surgical unit, all patients signed a written informed consent to utilize their biological material for scientific purposes. All patient samples were pseudonymized, and associated clinical data were obtained (age, sex, diagnosis; Supplementary Table 3).

### Proteomic analysis

#### Sample preparation for proteomic analysis

Fragments of CRC and normal tissues were resuspended in Lysis buffer (Tris 50 mM, Urea 6 M, SDS 4%, DTT 10 mM) and dissociated with a gentleMACS™ dissociator (Miltenyi Biotec) using the appropriate program for tissue homogenization. The homogenized samples were centrifuged at 13,000 rpm for 30 min at 4 °C, the supernatants were recovered, and a part subsequently processed for proteomic analysis.

#### Protein quantification and digestion

Protein quantification was achieved by the use of Qubit protein broad range assay kit (Thermo Fisher Scientific). Each sample was diluted with in Lysis buffer (Tris 50 mM, Urea 6 M, SDS 4%, DTT 10 mM) to have 20 µg of proteins in a volume of 22 µL and incubated 1 h at 37 °C with gentle agitation. Thus, disulphide bonds between cysteines were alkylated by adding 2.6 µL of 200 mM iodoacetamide (1 h incubation at 37 °C) and, finally, 0.4 µL of 100 mM DTT (20 min incubation at 37 °C). Then, 12 µL (corresponding to about 10 µg) were processed through Protein Aggregation Capture protocol [28]. Briefly, for each sample 5 µL (100 µg) of MagResyn Hydroxyl beads (Resyn Biosciences) were equilibrated by 2 sequential washes with 100 µL of 70% acetonitrile. Then, samples were added to the magnetic microparticles and the precipitation of proteins was obtained by the addition of acetonitrile to a final percentage of 70. The solution was incubated for 10 min at 1100 rpm. Three washes with 200 µL of acetonitrile were followed by one wash with 70% ethanol. Finally, the beads were resuspended in 50 µL of 50 mM triethylammonium bicarbonate. Trypsin-Lys C protease mix (Thermo Fisher Scientific) was added at a E/S 1/50 (200 ng). After overnight incubation (37 °C, 1100 rpm), the peptide solution was harvested, and residual peptides were recovered by washing the beads with 50 µL of 0.1% formic acid.

#### Strong cation exchange purification

Thirty µL of the digested peptides were purified by Strong Cation Exchange purification [29]. Briefly, the resin was sequentially conditioned and equilibrated by wash A (20% acetonitrile/ 0.5% formic acid) and wash B (80% acetonitrile/ 0.5% formic acid). Peptide mixture was then loaded after being diluted to 200 µL in wash B. Thus, were performed two washes (wash B, wash A). Peptides were eluted by adding 10 µL of the eluent solution (500 mM ammonium acetate/20% acetonitrile). Peptides were then lyophilized and resuspended in 30 µL of solution A (2% acetonitrile/0.1% formic acid).

#### nLC-MS/MS

Mass spectrometric analysis was carried out on a Orbitrap Exploris 480 mass spectrometer (Thermo Fisher Scientific) operating in positive ion mode, coupled with an Easy LC 1200 nanoscale liquid chromatography system (Thermo Fisher Scientific). The analytical column was a 17 cm pulled silica capillary (75 μm i.d.) packed in-house with 3 μm C18 silica particles (Dr. Maisch). Nanoelectrospray (nESI) was obtained by applying a potential of 2000 V on the column front-end through a tee piece. A binary gradient was used in order to obtain peptide elution. In particular, mobile phase A contained 2% acetonitrile/ 01% formic acid (v: v) while mobile phase B was composed of 80% acetonitrile/ 01% formic acid. The flow rate was set to 230 nl/min. The total gradient duration was of 55 min. In particular, the mobile phase B ramped from 6 to 12% in 11 min, from 12 to 40% in 29 min and from 40 to 100% in 8 min. Then, was maintained 5 min at 100%. The analytical column was equilibrated at 0% mobile phase B for 2 min. Data-independent acquisition enclosed 30 windows with a full scan at resolution of 60,000 (AGC target Standard and maximum injection time auto) and 30 DIA scans with a resolution of 30,000 (AGC target of 5e5, maximum injection time of 50 ms and normalized collision energy of 25). In detail, isolation window was set in order to have 23 windows of 15 m/z, 4 windows of 30 m/z and 3 windows of 50 m/z. The overlap was equal to 0.5 m/z. The resulting m/z range was 350–1010.

#### Data analysis and results reporting

Raw files were searched by Spectronaut software (version 18.7) against the database Human 1 Protein 1 Gene (20577 seq, March 2022). Analysis settings were left as default modifying only the following parameters: only peptides shared between the proteins of the same protein group (protein group specific) were considered for quantification. Quantification was performed on precursor identified in at least the 20% of runs. Missing values were imputed using background signal as imputation strategy. One CRC sample was excluded because it did not pass the quality control (a high level of blood-derived proteins was detected; this contamination prevented efficient sample normalization). The statistical analysis was employed as follows:

The report from Spectronaut was then loaded on Perseus v. 2.0.11.0. The analysis was performed following these steps: Protein Quantities were log2 transformed, the matrix was then filtered keeping only protein groups quantified in at least 4 samples of a group. Then, significant differentially expressed proteins were identified by applying a two-sample test with a Permutation based FDR set at 0.05 (S0 was set to 0.2).

### Pathway analysis

Gene set enrichment analysis (GSEA) was performed in preranked mode using the GSEA tool (https://www.gsea-msigdb.org/gsea/index.jsp) with gene sets from the HALLMARK, Wikipathways, and REACTOME collections. Heat maps were generated using the Heatmapper tool [30]. Molecular Signature Database (MSigDB) Hallmark 2020 and Reactome2024 pathway analysis was performed using enrichr using the differentially expressed proteins (FDR < 0.05) in CRC tissues compared with NDT or NAT [31].

For identification of upstream regulators, IPA (QIAGEN) software was used. Protein differentially expressed between CRC and NDT were uploaded to IPA. Upstream Regulators were identified and filtered by absolute activation z-score > 2.

### Tissue immunohistochemical and immunofluorescence analysis

Immunostaining was performed on 4 Tissue Micro Array (TMA) slides (Tissue Array, # CO801a) including CRC and matched adjacent normal colon tissues (37 cases of adenocarcinoma, 2 cases of mucinous adenocarcinoma and 1 case of signet-ring cell carcinoma, duplicate cores per case).

Further we analyzed 40 CRC and matched normal tissue sections from our two independent patient cohorts.

Formalin-fixed, paraffin-embedded 2.5 μm sections of CRC, normal tissue adjacent to tumor (NAT), and normal tissue distant from tumor (NDT) were collected using a Diapath Galileo system and processed for fluorescence microscopy or immunohistochemistry. Immunohistochemistry was performed using a Dako OMNIS automated staining system. Heat-induced antigen retrieval was achieved automatically using EnVision FLEX Target Retrieval Solution. Immunohistochemical staining was performed using the following primary antibodies, P53 clone DO7, CD68 clone PDM1, PDPN clone D240, all ready to use.

For immunofluorescence analysis, deparaffinized and rehydrated sections of CRC and normal tissue samples were washed with DPBS and permeabilized with 0.1% Triton X-100 in DPBS for 30 min at room temperature, then washed with DPBS and subsequently blocked with 1% BSA in DPBS for 30 min at room temperature. Subsequently, the sections were incubated with primary antibodies against PDPN (Thermo Fisher Scientific, # MA5-16267, 1:100) and mouse monoclonal anti-α-SMA (Sigma Aldrich, A2547, 1:100) for 2 h at room temperature and then stained with appropriate Alexa Fluor-conjugated secondary antibodies (diluted 1:1200), for 1 h at room temperature following the manufacturer’s instructions. The sections were then washed with DPBS and stained with DAPI for counterstaining of nuclei. The images were obtained using a Fluoview FV3000 microscope (Olympus) and the images were taken with the FV31S-SW software.

### Imaging and scoring

Slides of tumor specimens were visualized using an Olympus BX41 microscope (Olympus), and the images were taken with CSV1.14 software, using a CAM XC-30 for image acquisition. Immunoreaction scores were assigned with a range of 0 (negative) to 4 (strongly positive) using a decimal number scale with progression in quarters. For each slide, a minimum of 100 cells were evaluated, and seven serial sections were scored by three expert pathologists according to the histopathological criteria of the World Health Organization (5th edition).

### Immunofluorescence of cultured cells

For immunofluorescence microscopy assays, MSCs were washed with PBS, fixed in 4% paraformaldehyde (PFA) for 20 min, permeabilized with 0.1% Triton X-100 for 5 min, and stained for immunofluorescence evaluation with the indicated antibodies, as previously described [32]. The coverslips were mounted onto glass slides with ProLong Gold antifade reagent (Invitrogen, P10144). Images were acquired with a Leica DMi8 inverted microscope and LasX software (v. 3.7.423463; Leica Microsystems CSM GmbH).

### Cell cultures

Human Mesenchymal Sromal Cells (hMSC) were purchased from Lonza (PT-2501) and grown in DMEM High Glucose (SIAL, DMEM-HPXA) supplemented with 1% penicillin-streptomycin (SIAL, PEN/STREP), 20 mM L-glutamine (SIAL, LGlu), 1X Non-Essential Aminoacids (Gibco, 1140-050) and 10% Mesencult Human Supplement (Stemcell Technologies, 05402) at 37 °C in a 5% CO_2_ fully-humidified atmosphere. All experiments were performed using early passage hMSCs, up to passage 5. For pharmacological treatment, hMSC cells were treated with: Verteporfin 250 nM for 48 h (Sigma Aldrich, SML0534); Stemolecule ALK5 inhibitor 10 µM for 48 h (Stem Cell Technologies, 73792); Y27632 10 µM for 48 h (Sigma Aldrich, SCM075); TGF-β1 10 ng/mL for 48 h (PeproTech, 100 − 21); IFN-γ 40 ng/mL for 48 h (PeproTech, 300-02); IL-6 40 ng/mL for 48 h (PeproTech, STDA 200-06); CY12-RP2 [33] was synthesized by GenScript Biotech with a purity confirmed to be > 98% by high-performance liquid chromatography (HPLC) and mass spectrometry (MS). CY12-RP2 was used at a concentration of 50 µM, as previously reported [33]. Caco-2, HCT116 and LS174T cells were purchased by ATCC and grown in DMEM High Glucose (SIAL, DMEM-HPXA) supplemented with 1% penicillin-streptomycin (SIAL, PEN/STREP), 20mM L-glutamine (SIAL, LGlu) and 10% Fetal Bovine Serum (Gibco, A5669701) at 37 °C in a 5% CO_2_ fully-humidified atmosphere.

### Cell transfection

hMSCs were transfected using Lipofectamine RNAiMAX according to the manufacturer’s protocol (Invitrogen, 13778075). Briefly, 10 µM gene-specific siRNA oligomers (ON-TARGETplus Human PDPN siRNA smartpool, Dharmacon, L-017560-01-0005) or a non-targeting control pool (ON-TARGETplus Non-targeting Control Pool, Dharmacon, D-001810-10-05) were diluted in Opti-MEM reduced serum medium (Gibco, 31985-047) and mixed with Lipofectamine. After 5 min of incubation at room temperature, the complexes were added to the cells. The final amount of siRNA used per well was 5 pmol (24-well) or 25 pmol (6-well). Cells were analyzed after 48 h from transfection.

### CD14^+^ peripheral blood monocytes isolation and macrophage differentiation

Peripheral blood was obtained from healthy donors after written informed consent and peripheral blood mononuclear cells (PBMCs) were isolated by density gradient centrifugation using Lymphocyte Separation Medium (SIAL, LSM-A) as previously described [34]. CD14^+^ monocytes were isolated from PBMCs by positive selection through magnetic-activated cell sorting (MACS) system using CD14-conjugated microbeads (Miltenyi Biotec, 130–050–201), according to manufacturer’s instructions. For differentiation toward the classically-M1 or alternatively-M2 activated macrophage phenotype, CD14^+^ monocytes were cultured in RPMI-1640 (SIAL, RPMI-A) supplemented with 1% penicillin-streptomycin and 10% FBS at 37 °C in a 5% CO_2_ fully humidified atmosphere. In order to differentiate cells into M1 or M2 macrophages, a “phased strategy” was used [35]. Specifically, for M1 macrophages differentiation, CD14 + cells were maintained in a medium containing granulocyte macrophage colony-stimulating factor (GM-CSF, PeproTech, 300-03) for 5 days. On the sixth day, the medium was renewed, and cells treated with a cocktail of GM-CSF, IFNγ (PeproTech, 300-02), IL-6 (PeproTech, 200-06), and lipopolysaccharide (Sigma Aldrich, L2018) for 4 more days. For M2 macrophage differentiation, CD14 + cells were maintained in a medium containing colony-stimulating factor (M-CSF, PeproTech, 300 − 25) for 5 days, and then M-CSF, IL-4 (PeproTech, 200-04), IL-6, and IL-13 (PeproTech, 200 − 13) for additional 4 days. All the cytokines were used at a concentration of 20 ng/mL. Macrophage differentiation was routinely characterized by qRT-PCR (Supplementary Fig. 5).

### Co-cultures

#### hMSC-macrophage indirect co-cultures

The hMSC (1 × 10^4^/cm^2^) were seeded in 12 or 24-well plate. After 24 h, differentiated M1 or M2 macrophages (5 × 10^3^/cm^2^) were seeded into the upper chamber of a Transwell permeable support (0.4 μm polyester membrane, Costar, 3470/3460) in order to create an indirect co-culture condition that allowed macrophage-produced molecules to interact with the hMSC in the lower chamber. After 48 h, macrophages were discarded and hMSC analyzed or used as a feeder for colon adenocarcinoma cell growth.

#### hMSC-colon adenocarcinoma cell direct co-cultures

The hMSC (1 × 10^4^/cm^2^) were seeded in 12 or 24-well plate. After 24 h, hMSC were treated, transfected or exposed to macrophage indirect co-culture. After 48 h, the medium was replenished and colon adenocarcinoma cells (5 × 10^3^/cm^2^) were seeded in direct co-culture on pre-conditioned hMSC. After 48 h, the size of colon adenocarcinoma cell colonies was evaluated.

### Western blot

hMSCs were lysed for 30 min at 4 °C in HEPES-glycerol lysis buffer (50 mM HEPES, pH 7.4, 150 mM NaCl, 10% glycerol, 1% Triton X-100 (Sigma Aldrich, T9284), 1.5 mM MgCl2, 1 mM EGTA) containing 1 µg/mL leupeptin (Sigma Aldrich, L2884) and 1 µg/mL aprotinin (Sigma Aldrich, A1153). Samples were clarified by centrifugation at 15,700 × g at 4 °C for 15 min. Laemmli sample buffer was then added to supernatants. Samples were heated at 95 °C for 3 min, separated by electrophoresis on 4–15% Mini-PROTEAN^®^ TGX™ precast protein Gels (Bio-Rad, 4561084DC) then transferred to polyvinylidene fluoride membranes (Bio-Rad, 1704272). Membranes were probed with primary antibodies, washed three times with PBS and Tween 0.1% (Bio-Rad, 1610781), and incubated with peroxidase-conjugate secondary antibodies. Membranes were visualized using Immobilon western chemiluminescent horseradish peroxidase substrate (Millipore, WBKLS0), images were acquired by UVITEC Alliance Mini HD9 (UVitec, U.K.), and the protein levels detected were quantified using UVITEC NineAlliance 1D software.

### RT-qPCR

Reverse transcription was performed in a final volume of a 20-µL reaction using SensiFAST cDNA Synthesis Kit (Meridian Bioscience, BIO-65054) according to the manufacturer’s instructions. The primers used are listed in Supplementary Table 1. All cDNA samples were diluted up to three times with ddH2O and amplified in triplicate with 200 nM of each primer and SensiFAST SYBR Hi-ROX (Meridian Bioscience, BIO-92020). Quantitative polymerase chain reaction (qPCR) analyses were conducted in real-time using a QuantStudioTM 7 Pro Real-Time PCR System (Applied Biosystem). Gene expression was normalized to the b2-microglobulin (B2M), b-actin (ACTB) and Histone H3.3 housekeeping genes. Analysis was conducted using the comparative cycle threshold (Ct) method.

### Chromatin immunoprecipitation (ChIP)

ChIP-qPCR assays from tissue are performed as previously described [36]. Briefly, cancer and matched healthy tissues from three CRC patients were fixed using formaldehyde to a final concentration of 1% and under rotation at room temperature for 15 min. DNA from lysed nuclei was sheared to 600–800 bp fragments using a Bioruptor sonicator (Diagenode). About 80 µg of sheared chromatin and 5 µg of antibody plus 40 µl of magnetic beads (Dynabeads^®^ Protein G 10004D Thermo Fisher Scientific) were used for each immunoprecipitated sample. Mouse anti-YAP1 (Abcam, ab205270), mouse anti-TAZ (Abcam, ab224239), Mouse anti-Histone H3 (acetyl K27) ChIP Grade (Abcam, ab4729), mouse anti-IgG (Santa Cruz, sc-2025) are used.

Quantitative real-time PCR was carried out with a QuantStudio 5 Fast Real Time PCR Applied Biosystems (Thermo Fisher Scientific) using SYBR RT-qPCR Master Mix (Applied Biosystems). The amount of immunoprecipitated DNA in each sample was calculated as the fraction of the input [amplification efficiency^(Ct INPUT–Ct ChIP)^] (Input 1:100 dilution). The TEAD binding region A and B identified onto the PDPN promoter were amplified using the primer pairs included in Supplementary Table 1. All reactions of PCR from each point were performed in biological triplicate.

### Public repository of patient datasets and bioinformatic tools

We explored the following patient datasets: TCGA of colon and rectal adenocarcinoma [37]; GTex expression data of normal tissues [38]; Sidra-LUMC AC-ICAM: an atlas and compass of immune-cancer-microbiome interactions [39]; TCGA Colorectal Adenocarcinoma Firehose Legacy.

The bioinformatics tools that were used to generate the analyses by exploring the above datasets are: GEPIA2 differential gene expression analysis (http://gepia2.cancer-pku.cn/#index), the cutoff for *p* values was 0.05; TIMER (https://cistrome.shinyapps.io/timer/) for the analysis of cancer immune infiltration and allows users; cBioPortal (www.cBioportal.org) to obtain gene expression data and to analyze multi-dimensional cancer genomic data.

### Statistics

Values are expressed as mean ± standard deviation (SD). A paired or unpaired 2-tailed Student’s t test was used for comparisons between two groups. For comparisons of 1 factor across multiple groups, one-way ANOVA was performed followed by the post-hoc Tukey test. For comparisons of 2 factors across multiple groups, two-way ANOVA was performed followed by the post-hoc Tukey test. Correlations were performed by the Pearson correlation method. GraphPad Prism 8 (GraphPad Software) was used for statistical analysis and graphing. Values of *p* < 0.05 were considered statistically significant. All experiments were independently replicated at least 3 times.

## Results

### Proteomic profile of CRC tissues

**Fig. 1:**
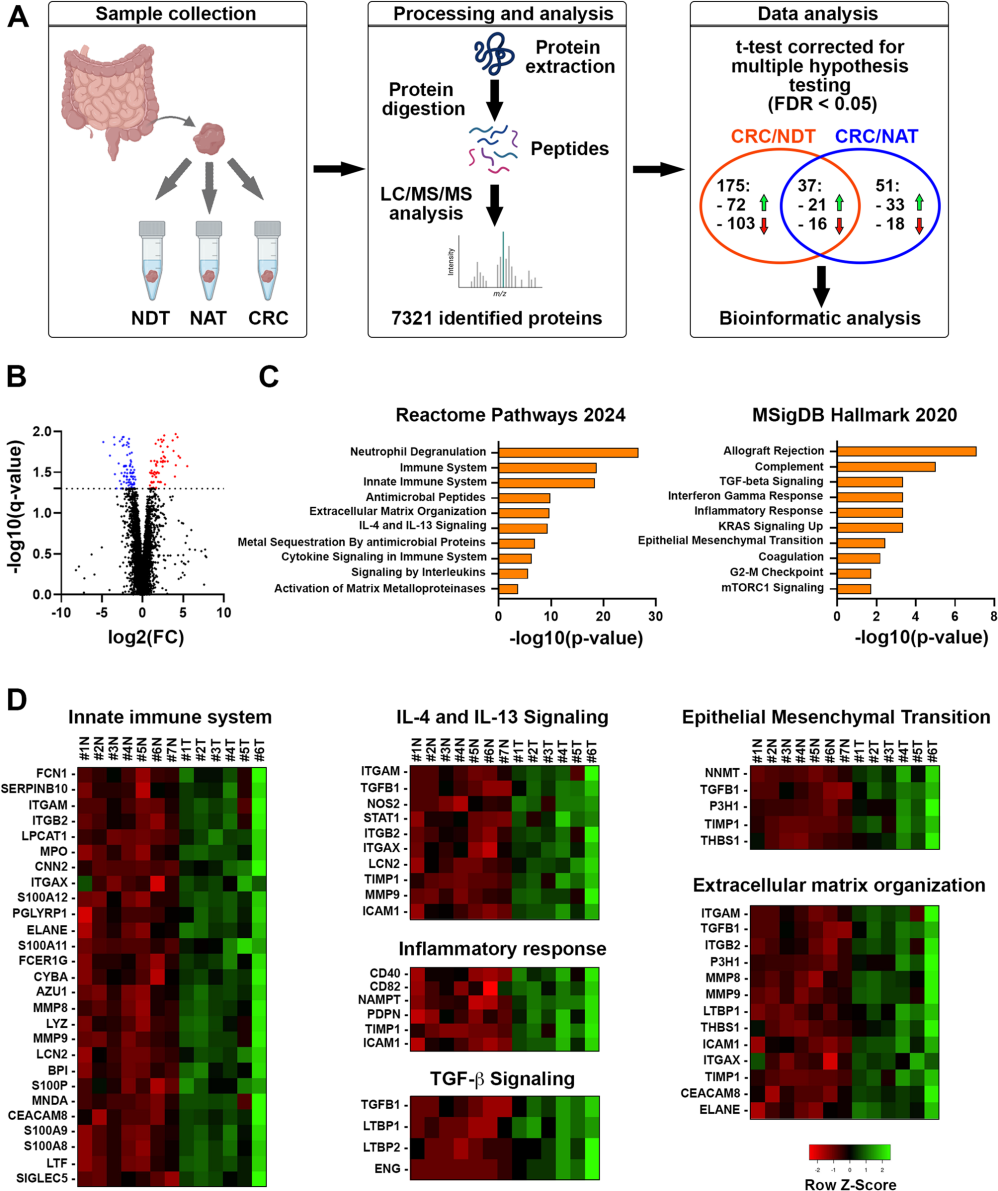
Pathways associated with significantly upregulated proteins in CRC compared to matched healthy tissue. **(A)** Label-free LC–ESI–MS/MS experimental design and main results. Image created with BioRender. **(B)** Volcano plot displaying the log 2 fold change (x axis) against the − log 10 statistical q value (y axis) for all proteins differentially expressed between NDT and CRC tissues. Proteins with significantly decreased levels in CRC (q < 0.05) are shown in blue, while the proteins with significantly increased levels in CRC are noted in red. **(C)** Enrichment pathway analysis of significantly up-regulated proteins in CRC versus NDT from the REACTOME 2024 and MSigDB Hallmark 2020 databases. **(D)** Heatmap showing protein expression data of innate immune system, IL-4 and IL-13 signaling, inflammatory response, TGF-b signaling, EMT and ECM organization. Green and red colors refer to the z-score of the data by row

LC-ESI-MS/MS was used to assess changes in protein abundance among patient matched primary CRC, normal tissue adjacent to the tumor (NAT) and normal tissue distant to the tumor (NDT) (Fig. 1A). Overall, analyses revealed 175 (72 up and 103 down) significantly changed proteins in CRC versus NDT and 51 (33 up and 18 down) significantly changed proteins in CRC versus NAT over 7321 identified proteins (Fig. 1A-B, Supplementary Fig. 1, Supplementary Table 4). We performed pathway enrichment analysis associated with the differentially expressed proteins. Among the top ten enriched pathways in both CRC versus NAT and CRC versus NDT comparisons associated with upregulated proteins, we identified several pathways related to the TME, such as innate immune system, IL-4 and IL-13 signaling, inflammatory response, TGF-β signaling, EMT, and ECM organization (Fig. 1C-D, Supplementary Fig. 2). Proteins downregulated in CRC tissues compared to NDT were mainly enriched in pathways related to cell metabolism, in particular of aerobic respiration and oxidative phosphorylation (Supplementary Fig. 2).

### PDPN is increased in the stroma surrounding CRC

PDPN has been previously shown to be upregulated in a variety of cancers including breast cancer, lung cancer, pancreatic cancer. Recently, clinicopathological results showed that high PDPN expression was significantly related to the poor prognosis of CRC patients [25, 40].

**Fig. 2:**
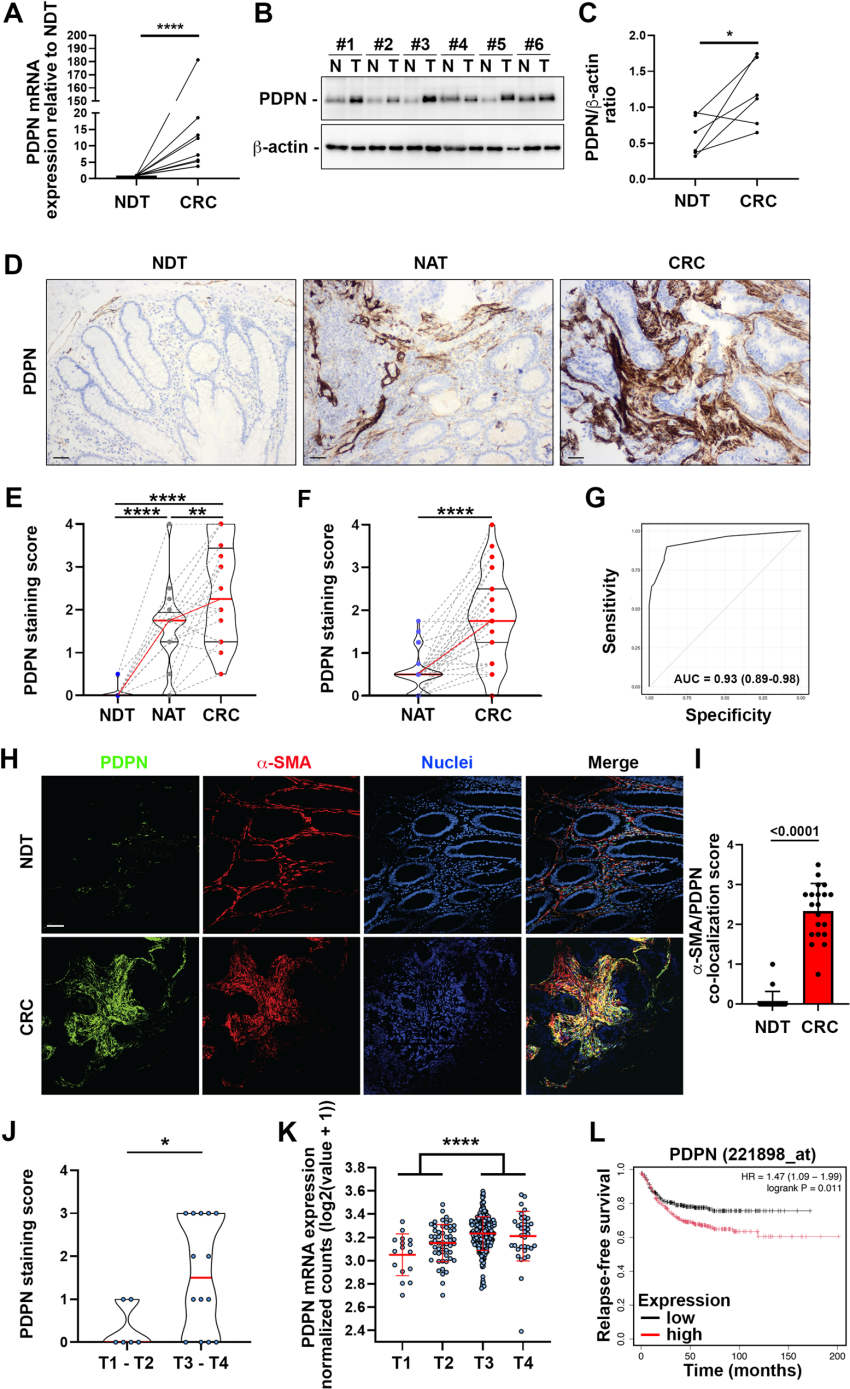
PDPN is increased in the stroma surrounding CRC. **(A)** RT-qPCR analysis of PDPN mRNA expression in matched normal tissue distant to the tumor (NDT) and CRC samples. Mann Whitney test, *p* < 0.0001, *n* = 9. **(B)** Western blot analysis of PDPN protein expression in matched NDT and CRC samples. β-actin was used as loading control. **(C)** The intensity of PDPN/β-actin ratio measured by densitometry is shown. Upaired t-test, *p* = 0.0199, *n* = 6. **(D)** Representative immunohistochemistry staining of PDPN in matched NDT, normal tissue adjacent to the tumor (NAT) and CRC tissues. Scale bar is 50 μm. **(E)** PDPN immunohistochemistry staining score was compared between NDT, NAT and CRC tissues. One-way ANOVA, *n* = 20. **(F)** PDPN immunohistochemistry staining score in NAT and CRC tissues from the tissue microarray. Paired t-test, *n* = 40. **(G)** Receiver Operating Characteristic (ROC) curve representing PDPN score sensitivity and specificity for the classification of tumoral vs. normal tissue. **(H)** Representative immunofluorescence staining of PDPN and a-SMA in CRC and NDT tissues. Scale bar is 50 μm. **(I)** Quantification of PDPN and a-SMA co-localization rates in normal and CRC tissues. Paired t-test, *n* = 20. **(J)** PDPN immunohistochemistry staining score in CRC tissues of patients with different tumor size. Unpaired t-test, *n* = 6 T1-T2, *n* = 14 T3-T4. **K)** PDPN mRNA expression in CRC tissues of patients with different tumor size. Data have been extracted from the Colon Sidra-LUMC AC-ICAM dataset [39]. Unpaired t-test, *n* = 69 T1-T2, *n* = 279 T3-T4. **L)** Kaplan-Meier survival analysis showing the relapse-free survival of patients with colon cancer stratified by the expression of PDPN. (adapted from KmPlot using the data from 1336 tumor samples from 16 independent cohorts) [43]. * *p* < 0.05, ** *p* < 0.01, *** *p* < 0.0001, **** *p* < 0.00001

Our proteomic analyses identified PDPN as one of the most upregulated proteins in CRC versus NDT tissue, with > 3 median fold change (Supplementary Fig. 3). RT-qPCR and western blot analysis of PDPN gene and protein expression in matched primary carcinoma and NDT, confirmed the proteomic data (Fig. 2A-C). The higher expression of PDPN in CRC versus normal tissues was further confirmed also at molecular level by analyzing GTex/TCGA colon and rectum adenocarcinoma public datasets (Supplementary Fig. 4). To investigate PDPN localization, we performed immunohistochemical staining on matched NDT, NAT and primary carcinoma of a cohort of 20 CRC patients. Analysis showed that PDPN was almost undetectable in NDT, where it marked lymphatic vessels as expected, but was significantly increased in CRC with a diffuse stromal staining (Fig. 2D-E). Of note, PDPN appeared significantly increased also in the lamina propria of NAT compared to NDT (Fig. 2D-E).

These findings were further validated using a CRC tissue microarray comprising a cohort of 40 matched NAT and primary carcinoma. The immunohistochemical analysis revealed a strong and significant increase in stromal PDPN in the primary carcinoma compared to the normal tissue (Fig. 2F). The accuracy of PDPN immunohistochemical staining to distinguish between tumoral and normal tissue was further tested by constructing ROC curves (Fig. 2G). PDPN has previously been shown to mark CAF in the stroma surrounding the CRC [41, 42]. We further confirmed the stromal origin of PDPN staining observed in the *lamina propria* surrounding CRC in our patient cohort, by co-staining PDPN with the CAF marker a-SMA (Fig. 2H). Confocal microscopy analysis revealed a significant association between PDPN and a-SMA around CRC, whereas co-localization in normal tissue was markedly low (Fig. 2I). Importantly, in a separate independent cohort of 20 CRC patients, we found that high expression of PDPN was associated with tumor size (Fig. 2J). This data was further confirmed at the molecular level by analyzing the Colon Sidra-LUMC AC-ICAM dataset (Fig. 2K). Moreover, the analysis of a colon cancer database including 1336 tumor samples from 16 independent cohorts, revealed that PDPN expression was significantly correlated with shorter relapse-free survival (Fig. 2L) [43]. Taken together, these results point to PDPN expression in CRC as a negative prognostic factor and suggest its potential role in the tumor microenvironment.

### CD68 + macrophages are increased in the tissue surrounding CRC and is associated to PDPN

**Fig. 3:**
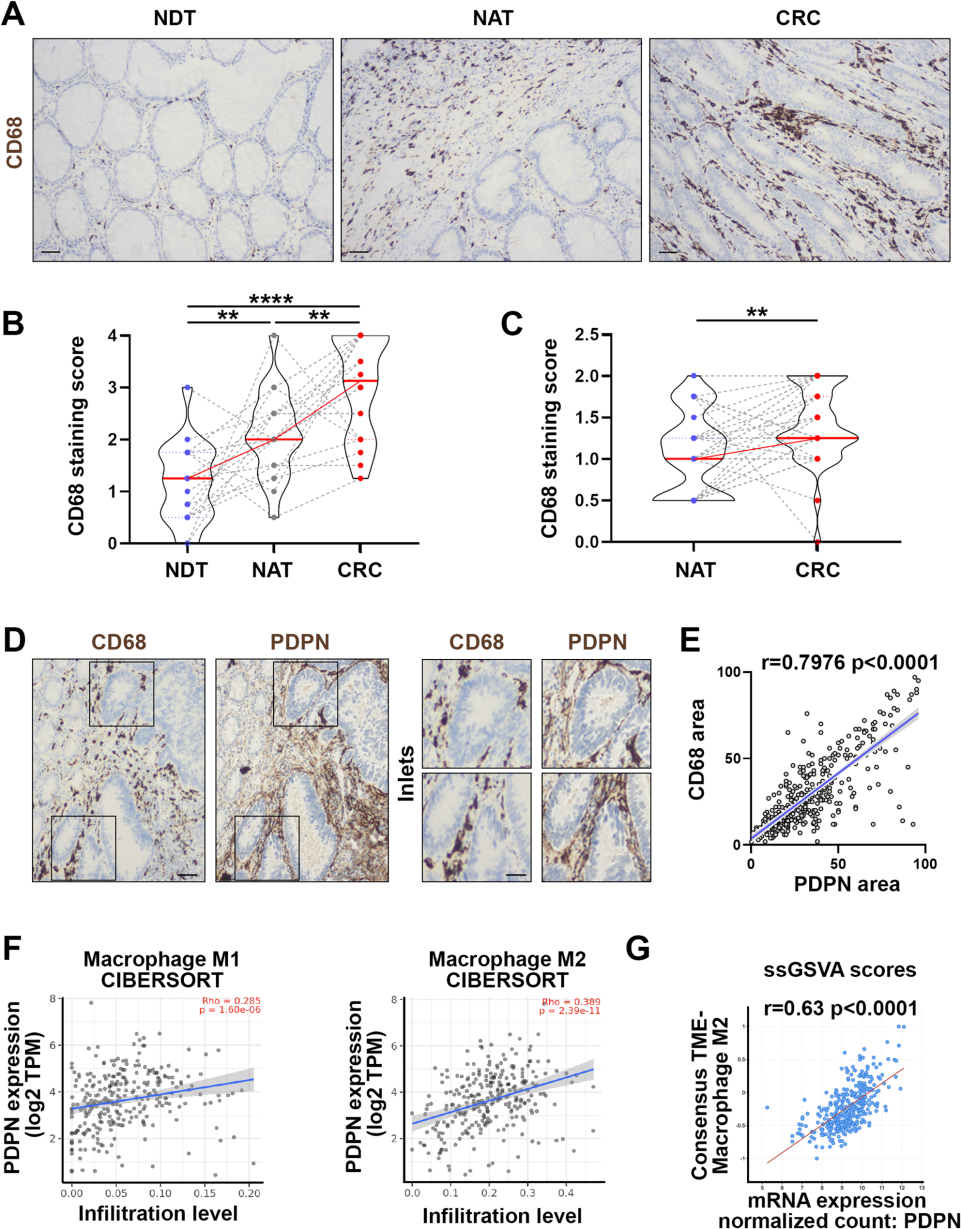
CD68 + macrophages are increased in the tissue surrounding CRC and are associated to increased stromal PDPN. **(A)** Representative immunohistochemistry staining of CD68 in matched NDT, NAT and CRC tissues. Scale bar is 50 μm. **(B)** CD68 immunohistochemistry staining score was compared between NDT, NAT and CRC tissues. One-way ANOVA, *n* = 20 **(C)** CD68 immunohistochemistry staining score in NAT and CRC tissues from the tissue microarray. Paired t-test, *n* = 40. **(D)** Representative PDPN and CD68 immunohistochemistry staining on consecutive serial sections of CRC tissues. Scale bar is 50 μm, inlet scale bar is 25 μm. **(E)** Pearson correlation analysis of PDPN and CD68 staining area coverage in CRC tissues. Data have been obtained from tissues deriving from 8 patients. A minimum of 30 randomly selected ROI per patient have been analyzed. **(F)** Pearson correlation analysis of PDPN mRNA expression and macrophage M1 and M2 infiltration level in CRC samples. Data derive from the CRC TCGA datasets analyzed by the CIBERSORT algorithm. **(G)** ssGSVA from Colon Sidra-LUMC AC-ICAM dataset showing Pearson correlation analysis of PDPN and M2 macrophage signature. * *p* < 0.05, ** *p* < 0.01, *** *p* < 0.0001, **** *p* < 0.00001

Our proteomic analyses showed a significant enrichment of proteins related to innate immunity and to IL-4 and IL-13 signaling, well-known inducer of macrophages differentiation. To validate these data, we performed immunohistochemical staining of the macrophage marker CD68 in our cohort of 20 matched NDT, NAT and primary CRC. The analysis confirmed a significant increase in CD68 + macrophage staining in the *lamina propria* surrounding tumors compared to normal tissues (Fig. 3A-B). Importantly, similar to PDPN, we observed a marked increase in CD68 + macrophage staining also in the NAT compared to the NDT (Fig. 3A-B). These findings were further validated using the CRC tissue microarray with matched NAT (Fig. 3C). Of note, the analysis of consecutive CRC tissue sections from our patient cohort revealed a well-defined spatial correlation between CD68 + macrophages and stromal PDPN staining (Fig. 3D-E), suggesting that PDPN stromal expression is linked to the extension of macrophage infiltration. These data were further confirmed at a molecular level, filtering the TCGA expression data with the CIBERSORT algorithm, we observed that PDPN mRNA levels were significantly associated with the molecular signature identifying classically and alternatively activated macrophage subpopulations, with a more significant association for the latter (Fig. 3F). Further, ssGSVA dataset interrogation showed a strong positive correlation between PDPN gene expression and alternatively activated macrophages (Fig. 3G).

### Macrophage secreted factors increase PDPN expression in stromal cells

**Fig. 4:**
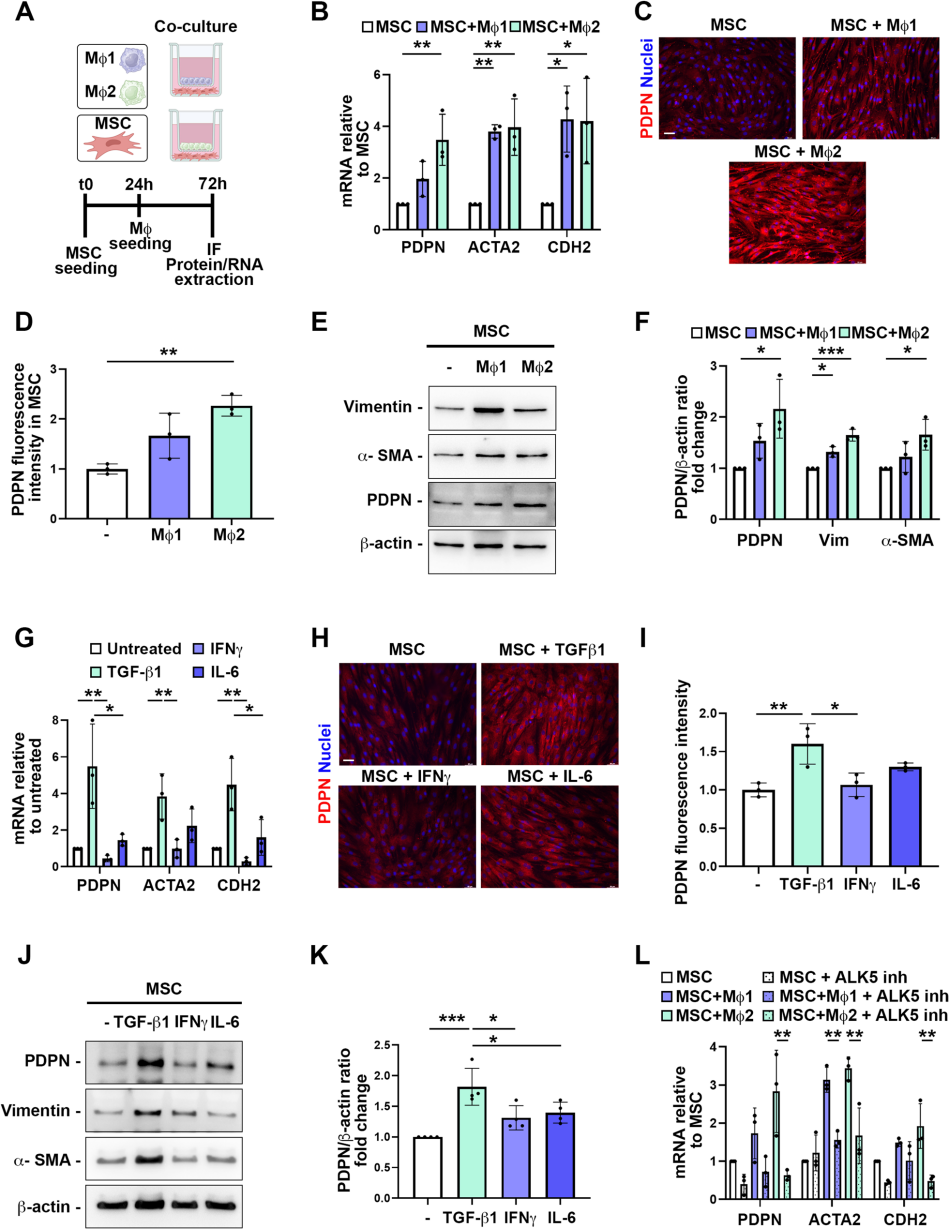
Alternatively activated macrophages increase PDPN expression in stromal cells. **A**) Set up of indirect macrophages (Mφ)/mesenchymal stromal cells (MSC) co-cultures. Image created with BioRender. **B**) RT-qPCR analysis of PDPN, ACTA2 (a-SMA) and CDH2 (N-Cadherin) mRNA expression in MSC cultured alone or co-cultured with classically activated M1 or alternatively activated M2 macrophages. One-way ANOVA, *n*=3. **C**) Representative immunofluorescence staining of PDPN in MSC cultured alone or co-cultured with classically activated M1 or alternatively activated M2 macrophages. Nuclei were counterstained with Hoechst. Scale bar is 50 mm. **D**) Quantification of PDPN staining intensity of MSC treated as in C. One-way ANOVA, *n*=3.  **E**)Western blot analysis of cancer associated fibroblast markers Vimentin, a-SMA and PDPN in MSC cultured alone or co-cultured with classically activated M1 or alternatively activated M2 macrophages. β-actin was used as loading control. **F**) The intensity of PDPN, vimentin or a-SMA/β-actin ratio measured by densitometry is shown. One-way ANOVA, *n*=3. **G**) RT-qPCR analysis of PDPN, ACTA2 and CDH2 mRNA expression in MSC cultured alone or in presence of 10 ng/mL TGF-β1, 40 ng/mL IFNγ or 40 ng/mL IL-6. One-way ANOVA, *n*=3. **H**) Representative immunofluorescence staining of PDPN in MSC cultured alone or in presence of 10 ng/mL TGF-β1, 40 ng/mL IFNγ or 40 ng/mL IL-6. Scale bar is 50 mm. **I**) Quantification of PDPN staining intensity of MSC treated as in I. One-way ANOVA, *n*=3. **J**) Western blot analysis of cancer associated markers Vimentin, a-Sma and PDPN in MSC cultured alone or in presence of 10 ng/mL TGF-β1, 40 ng/mL IFNγ or 40 ng/mL IL-6. **K**) The intensity of PDPN/β-actin ratio measured by densitometry is shown. One-way ANOVA, *n*=3. **L**) RT-qPCR analysis of PDPN, ACTA2 (a-Sma) and CDH2 (N-Cadherin) mRNA expression in MSC cultured alone or co-cultured with classically activated M1 or alternatively activated M2 macrophages in presence or not of 10 μM TGF-β1 receptor (ALK5) inhibitor. One-way ANOVA, *n*=3. * *p* < 0.05, ** *p* < 0.01, *** *p* < 0.0001.

To study a possible role of macrophage infiltration in CRC as a driver of stromal PDPN expression, we established indirect co-culture systems. Classically and alternatively activated macrophages were obtained by differentiating peripheral-blood derived monocytes by a phased strategy (Supplementary Fig. 5) [35]. Differentiated macrophages and mesenchymal stromal cells (MSCs) were plated in trans-well co-culture systems (0.4 μm pores) on the insert and on the bottom chamber well respectively (Fig. 4A). After 48 h, analyses of the co-cultures revealed an increased expression of PDPN mRNA and protein in MSCs, as shown by RT-qPCR, immunofluorescence and western blot. This upregulation occurred following co-cultures with both classically and alternatively activated macrophages, with a more pronounced effect exerted by the latter (Fig. 4B-F). Intriguingly, co-culture of MSC with the colon adenocarcinoma cell line Caco-2 also led to an increase of PDPN protein expression by MSCs, although to a lesser extent than when MSCs were co-cultured with alternatively activated macrophages (Supplementary Fig. 6). Concomitantly, we observed an increase of typical CAF markers, Vimentin, a-SMA and CDH2 in MSCs co-cultured with macrophages, in particular with the alternatively activated (Fig. 4F). Upstream regulator analysis using IPA software identified TGF-β1, IFN-γ, and TNF among the top ten upstream factors predicted to regulate the proteins altered in our proteomic analysis (Supplementary Fig. 7). Previous studies have shown in keratinocytes a positive regulation of PDPN expression by TGF-β1, IL-6 and IFN-g through TGF-β receptor-Smad pathway and JAK-STAT pathway, respectively [44]. Classically and alternatively activated macrophages are well-known sources of IL-6, IFN-g and TGF-β1, respectively. We thus investigated whether these macrophage-produced cytokines might modulate PDPN expression in MSCs. Our results demonstrated that TGF-β1 stimulated PDPN gene and protein expression in MSCs at various level (Fig. 4G-K). As expected, the increase of PDPN expression obtained by TGF-β1 treatment was accompanied by the increase of vimentin, CDH2 and a-SMA expression levels (Fig. 4G-J). Importantly, the inhibition of the activin receptor-like kinase 5 (ALK5) protein which is the TGF-β1 receptor, counteracted the increase of PDPN expression, as well as those of the CAF markers ACTA2 and CDH2, obtained by the co-culture with macrophages (Fig. 4L). Further supporting this finding, we found a positive correlation between the ssGSVA mRNA expression signature of TGF-β1 and PDPN mRNA expression in patients (Supplementary Fig. 8A).

### Macrophage and TGF-β1 induced PDPN stromal expression through YAP/TAZ

**Fig. 5:**
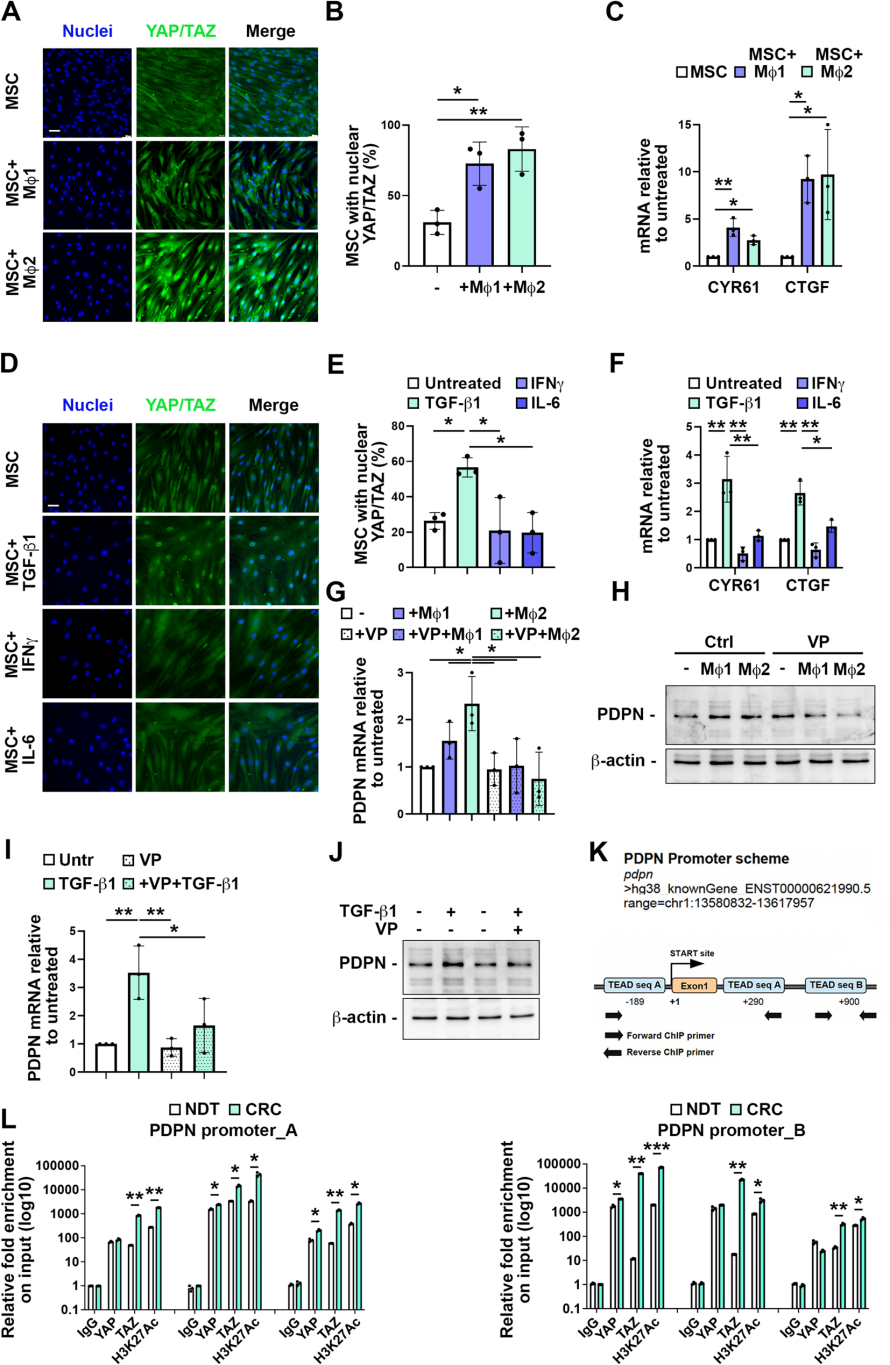
Macrophages and TGF-β1 induced PDPN stromal expression through YAP/TAZ. **(A)** Representative immunofluorescence staining of YAP/TAZ (green) in MSC cultured alone or co-cultured with classically activated M1 or alternatively activated M2 macrophages. Nuclei were counterstained with Hoechst. Scale bar is 50 μm. **(B)** Percentage of MSC displaying preferential nuclear YAP/TAZ localization. One-way ANOVA, *n* = 3. **(C)** RT-qPCR of YAP/TAZ targets CYR61 and CTGF in MSC cultured alone or co-cultured with classically activated M1 or alternatively activated M2 macrophages. One-way ANOVA, *n* = 3. **(D)** Representative immunofluorescence staining of YAP/TAZ (green) in MSC cultured alone or in presence of 10 ng/mL TGF-β1, 40 ng/mL IFNγ or 40 ng/mL IL-6. Nuclei were counterstained with Hoechst. Scale bar is 50 μm **(E)** Percentage of MSC displaying preferential nuclear YAP/TAZ localization. One-way ANOVA, *n* = 3. **(F)** RT-qPCR of YAP/TAZ targets CYR61 and CTGF in MSC cultured alone or in presence of 10 ng/mL TGF-β1, 40 ng/mL IFNγ or 40 ng/mL IL-6. One-way ANOVA, *n* = 3. **(G)** RT-qPCR of PDPN in MSC cultured alone or co-cultured with classically activated M1 or alternatively activated M2 macrophages in presence or not of 250 nM YAP/TAZ inhibitor verteporfin (VP). One-way ANOVA, *n* = 3. **(H)** Western blot analysis of PDPN protein expression in in MSC cultured alone or co-cultured with classically activated M1 or alternatively activated M2 macrophages in presence or not of 250 nM YAP/TAZ inhibitor verteporfin (VP). **(I)** RT-qPCR of PDPN in MSC cultured or not in presence of 10 ng/mL TGF-β1. Where indicated, MSC were pre-treated with 250 nM YAP/TAZ inhibitor verteporfin (VP). One-way ANOVA, *n* = 3. **(J)** Western blot analysis of PDPN protein expression in MSC cultured or not in presence of 10 ng/mL TGF-β1. Where indicated, MSC were pre-treated with 250 nM YAP/TAZ inhibitor verteporfin (VP). **(K)** Schematic representation of the PDPN gene promoter evaluated in the ChIP analysis. Light blue boxes represent TEAD-binding sites. +1 position indicates transcription start site. **(L)** The cross-linked chromatin purified by CRC and matched healthy tissues from three patients was used in ChIP experiments using the antibodies indicated in the figure. The TEAD binding sequences A and B (TEAD seq A and B) were analyzed by quantitative real time PCR. Normalization was performed to the amount of input chromatin. The ChIP samples were further tested by qPCR on a region that was negative for transcriptional factor recruitment. Bars represent mean ± SD from technical replicates. Unpaired t-test. * *p* < 0.05, ** *p* < 0.01, *** *p* < 0.0001

Previous studies suggested that *PDPN* gene expression might be regulated by the HIPPO pathway where YAP/TAZ are downstream cofactors, able to interact with TEAD family transcription factors in development and disease [45–47]. On these bases, we investigated whether the co-culture of MSCs with activated macrophages or treatment with related cytokines could influence the nuclear/cytoplasmic shuttling of YAP/TAZ. We observed a significant increase of MSCs with a prevalent nuclear YAP/TAZ localization when co-cultured with both classically and alternatively activated macrophages (Fig. 5A-B). The up-regulation observed by RT-qPCR of direct YAP/TAZ targets, CYR61 and CTGF, confirmed their transcriptional activation by macrophage released factors (Fig. 5C). The HIPPO pathway activity is best characterized by a YAP/TAZ transcriptional target gene signature of 29 genes [48]. Notably, we observed that PDPN expression was strongly correlated with the overall YAP/TAZ gene signature in the CRC TCGA dataset (Supplementary Fig. 8B).

Among the tested cytokines released by macrophages, TGF-β1 induced a robust and significant increase in nuclear YAP/TAZ localization in MSCs (Fig. 5D-E), along with the up-regulation of CYR61 and CTGF (Fig. 5F). To directly test whether YAP/TAZ regulates PDPN expression, MSCs, co-cultured with classically and alternatively activated macrophages, were treated with the YAP/TAZ inhibitor verteporfin [49, 50]. This treatment hampered the increase of PDPN expression in stromal cells obtained by co-culture with macrophages at both transcript and protein level (Fig. 5G-H). Consistently, the pre-treatment of MSC with verteporfin prevented the increased PDPN expression obtained by TGF-β1 incubation (Fig. 5I-J).

The link between YAP/TAZ co-transcriptional activity and the PDPN gene was further confirmed in three matched normal and CRC patient tissues by chromatin immunoprecipitation (ChIP) assays followed by qPCR analysis. Our data showed that TAZ, in particular, is recruited to TEAD-binding sequences on the PDPN promoter at a significantly higher level in CRC compared to the matched normal tissues (Fig. 5K-L). This binding correlated with increased acetylation at Lysine 27 of histone H3 indicating that PDPN in CRC tissues is more transcriptionally active than in healthy tissues (Fig. 5K-L).

### Stromal PDPN sustains colon adenocarcinoma cell growth

**Fig. 6:**
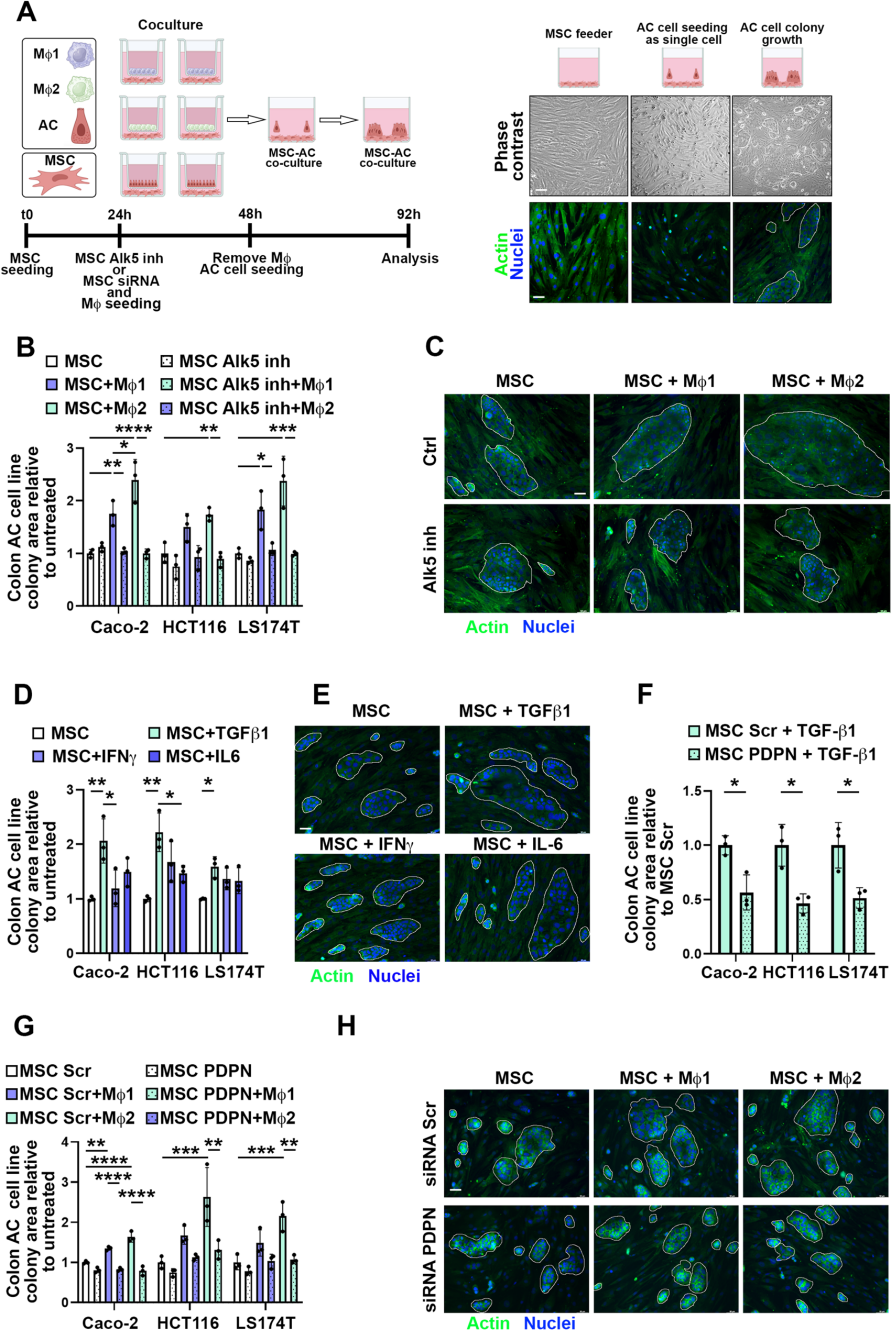
Stromal PDPN sustains colon adenocarcinoma cells growth. **(A)** Set up of indirect macrophages (MΦ)/mesenchymal stromal cells (MSC) co-cultures followed by direct MSC/colon adenocarcinoma (AC) cell co-culture. Image created with BioRender. MSC feeders were pre-conditioned or not with classically activated M1 or alternatively activated M2 macrophages. Where indicated MSC were pretreated with 10 µM TGF-β1 receptor ALK5 inhibitor or siRNAs. Colon AC cells were seeded as single cell suspension on MSC feeders and colony size measured after 48 h. **(B)** Colon AC cell colony size on MSC feeders pre-conditioned or not with classically activated M1 or alternatively activated M2 macrophages and in presence or not of 10 µM TGF-β1 receptor ALK5 inhibitor. One-way ANOVA, *n* = 3. **(C)** Representative immunofluorescence staining of actin (green) and nuclei counterstaining (blue) of MSC/Caco-2 co-cultures as in B. Scale bar is 50 μm. **(D)** Colon AC cell colony size on MSC pre-conditioned or not with 10 ng/mL TGF-β1, 40 ng/mL IFNγ or 40 ng/mL IL-6. One-way ANOVA, *n* = 3. **(E)** Representative immunofluorescence of actin (green) and nuclei counterstaining (blue) in MSC/Caco-2 co-cultures as in D. Scale bar is 50 μm. **(F)** Colon AC cell colony area after 48 h of direct co-cultures on MSC pre-conditioned with scramble (Scr) or PDPN specific siRNA and with 10 ng/mL TGF-β1. Unpaired t-test, *n* = 3. **(G)** Colon AC cell colony area after 48 h of direct co-cultures on MSC pre-conditioned with scramble (Scr) or PDPN specific siRNA and cultured alone or in indirect co-cultures with classically activated M1 or alternatively activated M2 macrophages. One-way ANOVA, *n* = 3. **(H)** Representative immunofluorescence of actin (green) in MSC/Caco-2 co-cultures as in G. Scale bar is 50 μm. * *p* < 0.05, ** *p* < 0.01, *** *p* < 0.0001, **** *p* < 0.00001

To assess the ability of PDPN overexpressing stroma to sustain colon adenocarcinoma cell growth, we established co-culture systems in which MSC, pre-conditioned with classically or alternatively activated macrophages, were used as a feeder for the growth of three different colon adenocarcinoma cell lines. Adenocarcinoma cells were seeded as single cell suspension on MSC feeders and after 48 h we observed the growth of typical colonies [51] (Fig. 6A). Our results showed that MSC feeders pre-conditioned with macrophages, particularly the alternatively activated, more effectively supported the growth of colon adenocarcinoma cells as evidenced by the larger colony area (Fig. 6B-C, Supplementary Fig. 9). Importantly, pre-treatment of MSC with ALK5 inhibitor, the TGF-β1 type I receptor inhibitor, abolished the effects of stromal pre-conditioning with macrophages on colon adenocarcinoma cell growth (Fig. 6B-C, Supplementary Fig. 9). We extended our investigation to assess whether the pre-conditioning of MSC with macrophage-produced cytokines could influence on colon adenocarcinoma cell growth. Hence, MSCs were pretreated for 48 h with TGF-β1, IL-6 or IFN-g and subsequently used as a feeder for tumor cell growth. As observed for PDPN gene and protein expression, TGF-β1 induced the most significant changes in stromal feeders supporting colon adenocarcinoma cell growth (Fig. 6D-E, Supplementary Fig. 10). To investigate the contribution of stromal PDPN upregulation in response to TGF-β1 treatment, we employed two distinct strategies: a pool of four combined PDPN-targeting siRNAs and a PDPN antagonist peptide CY12-RP2 [33]. Both treatments prevented the effects of TGF-β1 on MSC to sustain colon adenocarcinoma cell growth capacity (Fig. 6F, Supplementary Fig. 11). Further, PDPN-targeting siRNA suppressed also the effect of macrophage co-culture on adenocarcinoma cell growth (Fig. 6G-H, Supplementary Fig. 12). Overall, these results demonstrated that TGF-β1, secreted by alternatively activated M2 macrophages, induces modifications in the stroma that support colon cancer cell growth, highlighting PDPN as a key mediator of these tumor-promoting alterations.

### Stromal PDPN expression favors CAF activation and ECM synthesis

**Fig. 7:**
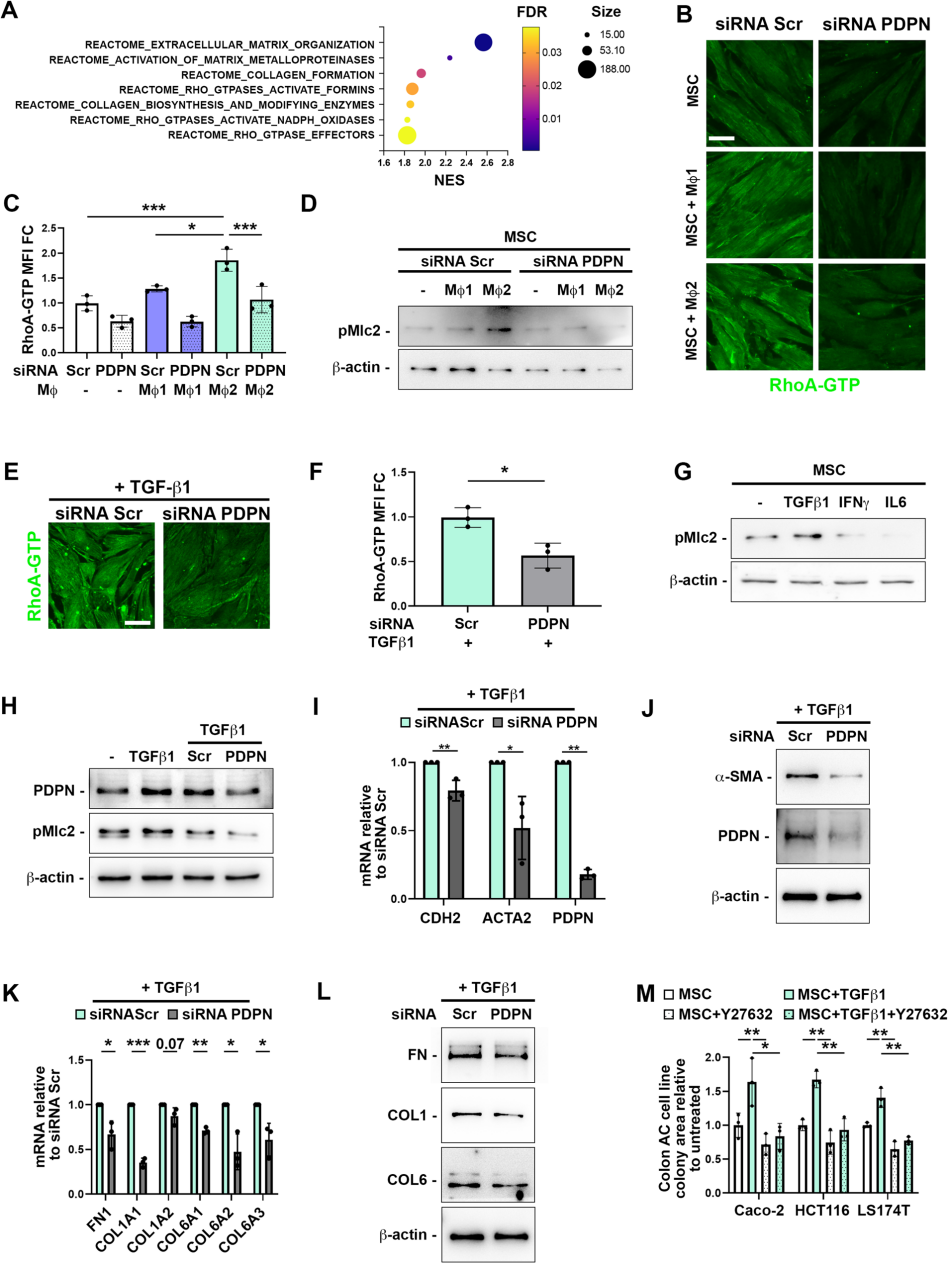
Stromal PDPN expression favors cancer associated fibroblast differentiation and extracellular matrix synthesis. **(A)** Normalized enriched score (NES) bubbleplot of GSEA results from tissue proteomics with MSigDB REACTOME genesets. **(B)** Immunofluorescence analysis of active RhoA-GTP in MSC treated with scamble (Scr) and PDPN specific siRNA and cultured alone or in indirect co-cultures with classically activated M1 or alternatively activated M2 macrophages. Scale bar is 20 μm. **(C)** Quantification of RhoA-GTP staining intensity of MSC treated as in B. One-way ANOVA, *n* = 3. **(D)** Western blot analysis of myosin light chain (Mlc2) phosphorylation in MSC cultured alone or in indirect co-cultures with classically activated M1 or alternatively activated M2 macrophages. **(E)** Immunofluorescence analysis of active RhoA-GTP in MSC treated with scamble (Scr) and PDPN specific siRNA and in presence of 10 ng/mL TGF-β1. **(F)** Quantification of RhoA-GTP staining intensity of MSC treated as in E. Unpaired t-test, *n* = 3. Scale bar is 20 μm. **(G)** Western blot analysis of Mlc2 phosphorylation in MSC cultured alone or in presence of 10 ng/mL TGF-β1, 40 ng/mL IFNγ or 40 ng/mL IL-6. **(H)** Western blot analysis of Mlc2 phosphorylation in MSC cultured alone or in presence of 10 ng/mL TGF-β1. Where indicated, MSC were treated with scramble (Scr) or PDPN specific siRNA. **(I)** RT-qPCR analysis of cancer associated fibroblast markers CDH2, ACTA2 and PDPN in MSC treated with scramble (Scr) or PDPN specific siRNA and 10 ng/mL TGF-β1. **(J)** Western blot analysis of a-Sma and PDPN in MSC treated with scramble (Scr) or PDPN specific siRNA and 10 ng/mL TGF-β1. **K)** RT-qPCR analysis of FN1, COL1A1, COL1A2, COL6A1, COL6A2, COL6A3 in MSC treated with scramble (Scr) or PDPN specific siRNA and 10 ng/mL TGF-β1. **L)** Western blot analysis of fibronectin (FN), type I collagen (COL1) and type VI collagen (COL6) in MSC treated with scramble (Scr) or PDPN specific siRNA and 10 ng/mL TGF-β1. **M)** Colon AC cell colony area after 48 h of direct co-cultures on MSC pre-conditioned with 10 ng/mL TGF-β1 and in presence or of 10 µM ROCK inhibitor Y27632. * *p* < 0.05, ** *p* < 0.01, *** *p* < 0.0001

Gene set enrichment analysis (GSEA) of our tissue proteomic data revealed among the most enriched REACTOME terms ECM organization, collagen biosynthesis and modifying enzymes and RHO GTPases effectors which are key regulators of cytoskeletal dynamics (Fig. 7A). We investigated whether macrophage-secreted factors could induce RhoA activation in MSC though indirect co-culture. Alternatively activated M2 macrophages significantly increased the RhoA-GTP level in MSC doubling the basal status (Fig. 7B-C). Importantly, the effect on RhoA activation was abrogated by PDPN siRNA treatment in MSCs (Fig. 7B-C). These results are in accordance with previous findings indicating that the cytoplasmic tail of PDPN is required for upregulation of RhoA activity [27]. RhoA activation leads to phosphorylation of its downstream effector, myosin light chain (MLC2), which is required for the assembly of actomyosin complexes and the promotion of actin–myosin-mediated contractile force generation. The increased activity of the Rho/ROCK/myosin pathway in MSCs induced by alternatively activated M2 macrophages was further supported by the increased MLC2 phosphorylation (Fig. 7D). Since it has been shown that TGF-β1-mediated EMT acts through a RhoA dependent mechanism [52], we investigated whether PDPN silencing could interfere with RhoA activation upon TGF-β1 treatment. As expected, TGF-β1 treatment increased the level of active RhoA-GTP. However, silencing PDPN via siRNA prevented the TGF-β1-induced RhoA activation, indicating the central role of PDPN in this mechanism (Supplementary Fig. 13 and Fig. 7E-F). Further, treatment of MSC with TGF-β1 induced MLC2 phosphorylation, which was counteracted by PDPN siRNA treatment (Fig. 7G-H). Of note, the increase of MLC2 phosphorylation was not observed when MSC were treated with the other macrophage-secreted cytokines tested (Fig. 7G). In parallel, siRNA-mediated reduction of PDPN expression or its inhibition by CY12-RP2 attenuated also the effects of TGF-β1 on the expression of CAF markers (CDH2 and a-SMA) and on ECM components (fibronectin, type I and type VI collagen), thereby confirming and extending the knowledge on the pivotal role of PDPN on the regulation of the EMT process (Fig. 7I-L, Supplementary Fig. 14). Finally, we explored whether the increased activity of the stromal RhoA/ROCK/myosin pathway could affect colon adenocarcinoma cell growth. Pre-conditioning of MSC with TGF-β1 in combination with the ROCK inhibitor Y27632, prevented the effect of TGF-β1 on the expression of CAF markers, on ECM components and on YAP/TAZ targets (Supplementary Fig. 15). This effect in turn resulted in the prevention of the enhanced tumor-supportive capacity of the stromal feeder observed with TGF-β1 treatment alone (Fig. 7M).

## Discussion

**Fig. 8:**
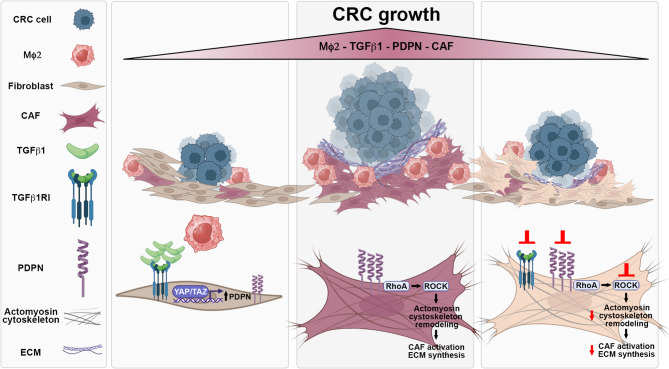
Role of stromal PDPN in CRC progression. During colorectal cancer (CRC) progression, tumor-associated macrophages (MΦ2) release soluble factors, including TGF-β1, which may induce upregulation of podoplanin (PDPN) in stromal cells via YAP/TAZ pathway activation. PDPN overexpression in these stromal cells promotes their differentiation into cancer-associated fibroblasts (CAF) by enhancing Rho/ROCK/myosin-dependent cytoskeletal remodeling and extracellular matrix (ECM) production. This, in turn, contributes to the establishment of a tumor-supportive microenvironment that facilitates CRC cell proliferation. Inhibition of PDPN expression in activated CAF, or treatment with either a TGF-β receptor inhibitor or a ROCK inhibitor, reduces their capacity to support the growth of colon adenocarcinoma cells

This study revealed that PDPN, a transmembrane glycoprotein enriched in CAF, is highly expressed in the TME of CRC and is associated with macrophage infiltration and tumor progression. By dissecting the molecular mechanisms driving PDPN expression in stromal cells, we found that it is tightly regulated by TGF-β1 secreted by alternatively activated M2-like macrophages and by YAP/TAZ pathway activation, which directly induces its transcription. Mechanistically, high PDPN expression in stromal cells upregulates CAF activation markers, enhances RhoA-mediated cytoskeletal contractility, and promotes ECM protein synthesis, thereby fostering a niche that supports colon adenocarcinoma cell growth (Fig. 8). Our findings expand the current knowledge on the complex interplay between stromal and immune cells in shaping the malignant CRC TME. Furthermore, our data highlight stromal PDPN as a promising negative prognostic biomarker and a potential therapeutic target for strategies aimed at suppressing the pro-tumorigenic functions of the TME in CRC.

Although both hereditary and sporadic CRCs have a well-defined genetic basis that triggers cancer initiation, modifications in the surrounding microenvironment play a critical role in tumor progression, dissemination, and response to treatment [53, 54]. The TME is a complex ecosystem in which cancer cells are surrounded by various non-malignant cell types, including immune cells and CAFs, embedded within an altered ECM. A comprehensive analysis of the crosstalk between tumor cells and the TME may reveal novel insights into tumor biology, identify therapeutic targets, and ultimately enable precision medicine approaches.

We employed LC-MS/MS to compare the proteomic expression profiles of CRC and matched healthy tissues to obtain an unbiased snapshot of the proteomic dynamics accompanying CRC progression. The proteome of CRC tissues revealed in particular diverse changes in TME-related pathways, primarily characterized by up-regulated proteins involved in immune system, macrophage differentiation, inflammation, EMT and ECM organization.

TAMs are the most abundant immune cells in the CRC TME [13]. While TAM infiltration is generally associated with poor prognosis in most solid tumors, its role in CRC remains controversial, particularly regarding the correlation between patient prognosis and the different TAM subtypes and their spatial distribution. Emerging evidence suggests that while high macrophage density may predict better prognosis in CRC patients undergoing adjuvant therapy, a high stromal density of M2-like macrophages is associated with worse cancer-specific survival [55–58]. Our proteomic analysis identified an enrichment of proteins linked to M2-like macrophages in CRC compared to matched healthy tissues. These factors include ARG1, TGFB1, MMP9, TIMP1, CD40, ITGAM and the enrichment of the IL-4 and IL-13 signaling pathway, prototypical direct inducers of macrophage differentiation toward the M2-like phenotype [59]. Moreover, our data showed that high macrophage density, particularly of the M2-like subtype, is locally associated with increased stromal PDPN expression, which in turn correlates with more advanced cancer stages and reduced relapse-free survival. Interestingly the relationship between PDPN+, CAFs and TAM infiltration has been previously described also in lung adenocarcinoma [60].

PDPN is upregulated in a wide range of cancers, where it is overexpressed not only in tumor cells but also in stromal cells, particularly CAFs [24, 61]. In tumor cells, PDPN expression is generally linked to poor prognosis. In various malignancies, including breast, lung, and pancreatic adenocarcinomas, where PDPN is absent from tumor cells, the expression of PDPN in CAFs has been associated with metastasis, poor patient outcomes, and reduced overall survival [62–64]. The clinical impact of PDPN overexpression by CAF in CRC remains unknown. Few controversial studies have pointed out the potential correlation of PDPN expression and clinicopathological characteristics in CRC. A study conducted almost twenty years ago, showed the expression of PDPN in the stroma of CRC as a favorable prognostic marker [65]. However, in this study the net dichotomization between PDPN-positive and PDPN-negative cases may have masked the exact impact of this protein expression on disease progression. Two more recent studies have linked PDPN expression in CRC to angiogenesis, poor prognosis and metastasis [25, 40].

Through multiple signaling pathways, CRC cells reprogram TAM into an anti-inflammatory, cancer-promoting M2 phenotype, which in turn releases cytokines, chemokines, and matrix metalloproteinases that synergistically drive EMT, CAF activation, matrix remodeling, and vascularization [66, 67]. In particular, TAMs are a rich source of TGF-β1, which have been shown to drive a stromal program involved in CRC initiation and metastasis. Of note, high serum TGF-β1 levels in CRC patients correlates with poor clinical outcomes [68–70]. Our data demonstrated that PDPN overexpression in mesenchymal stromal cells is driven by TGF-β secreted by M2-like macrophages and highlighted PDPN role in sustaining and enhancing TGF-β-mediated EMT and ECM synthesis. Although it is still too early to determine the safety and efficacy success rate of the many promising ongoing clinical trials targeting TGF-β function in combination with conventional therapy, one emerging aspect is its dependency on cancer subtype [71–73].

A consensus gene expression–based subtyping classification system for CRC has identified four main molecular subtypes (CMS1-4). CMS4, which accounts for 23% of CRCs, is characterized by strong TGF-β activation, stromal invasion, angiogenesis, high M2-like macrophage infiltration, poor prognosis, and therapy resistance [6]. Beyond TGF-β signaling, a key hallmark of CMS4 CRCs is the activation of the HIPPO pathway effectors YAP and TAZ [74, 75]. Notably, PDPN expression is significantly higher in CMS4 CRCs compared to other subtypes (Supplementary Fig. 16) and positively correlated with the YAP/TAZ and TGF-β signature in CRC datasets (Supplementary Fig. 8A-B). The crosstalk between TGF-β and YAP/TAZ pathways and their cooperative role in maintaining CMS4 features has been previously suggested [76]. Our data demonstrate a direct interplay between TGF-β secreted by M2-like macrophages and YAP/TAZ-mediated transcriptional regulation in driving the expression of PDPN, CTGF, and CYR61, factors involved in TME remodeling. Furthermore, our data show that the reduced growth of colon adenocarcinoma cells was accompanied by decreased nuclear localization of YAP/TAZ when the cells were cultured on PDPN-silenced stromal feeders. These findings may suggest a dual role for the HIPPO pathway in CRC, promoting both the transition and activation of CAF on one side and the growth of cancer cells on the other (Supplementary Fig. 17). Additionally, we found that PDPN overexpression in stromal cells, shaped by M2-like macrophages, activates the RhoA/ROCK/myosin pathway, which promotes cell contractile activity and ECM remodeling [77]. In agreement with our findings, the activation of RhoA/ROCK signaling has been identified as a central mechanism by which PDPN + CAFs promote tumor formation in lung adenocarcinoma [78, 79]. This functional loop may collectively drive the formation of an inflamed, stiff, fibrotic microenvironment, recently identified as an hallmark of CMS4 CRCs and might also contribute to the further release of active TGF-β from its latent-binding complex [80, 81]. Concurrently, the sustained activation of CAFs and the increase of ECM deposition might also contribute to therapy-resistance mechanisms through cancer cell insulation or drug retention [82–85]. Given its central role in coordinating these pathways, PDPN represents a potential vulnerability in CRC. Our data suggest that counteracting PDPN expression in activated CAFs or treating them with a TGF-β receptor inhibitor or a ROCK inhibitor, reduces their ability to support colon adenocarcinoma cell growth. Future studies on complex 3D ex vivo models will certainly aim to determine whether interfering with PDPN-mediated stromal rearrangement could enhance drug response of CRC cells.

## Conclusions

In summary, this study enabled us to identify targets and generate testable hypotheses on the mechanism leading to the aberrant crosstalk between the stromal and the immune components of the CRC TME. Our results revealed that PDPN is a key player in the stromal program that sustains CRC progression and targeting its regulation and function might offer new opportunities to modulate the pro-tumorigenic functions of the TME.

## Supplementary Information

Below is the link to the electronic supplementary material.

Supplementary Material 1

Supplementary Material 2

Supplementary Material 3

Supplementary Material 4

Supplementary Material 5

## Data Availability

RAW LC-MS/MS data analyzed during the current study are available from the corresponding author on request.

## Abbreviations

CRC: Colorectal cancer
TME: Tumor immune microenvironment
EMT: Epithelial-mesenchymal transition
ECM: Extracellular matrix
CAFs: Cancer-associated fibroblasts
TAMs: Tumor-associated macrophages
MSCs: Mesenchimal stem cells
PDPN: Podoplanin
TGF-β: Transforming growth factor beta
YAP1: Yes-associated protein 1
TAZ/WWTR1: WW domaing-containing transcriptional coactivator 1
