# Supplementary material for: Proteomic profiling identifies a stromal TGF-β1/podoplanin axis as a driver of colorectal cancer progression
Source: J Exp Clin Cancer Res. 2025 Aug 22;44:247. doi: 10.1186/s13046-025-03496-3 (PMC12372361; doi:10.1186/s13046-025-03496-3)
Supplement: Supplementary file 1 — Supplementary Material 1 [file 13046_2025_3496_MOESM1_ESM.docx]

**Supplementary Table 1. Primer list**

| **Gene** | **Forward Primer** | **Reverse Primer** |
| --- | --- | --- |
| **CYR61** | AGCCTCGCATCCTATACAACC | TTCTTTCACAAGGCGGCACTC |
| **CTGF** | AGGAGTGGGTGTGTGACGA | CCAGGCAGTTGGCTCTAATC |
| **PDPN** | ACCAGTCACTCCACGGAGAA | GCGAGTACCTTCCCGACATT |
| **B-ACTIN** | GACAGGATGCAGAAGGAGATTACT | TGATCCACATCTGCTGGAAGGT |
| **H3** | GTCTCTGTACCATGGCTCGT | CAGTACCAGGCCTGTAACGA |
| **B2M** | TGCTGTCTCCATGTTTGATGTATCT | TCTCTGCTCCCCACCTCTAAGT |
| **CDH2** | CGGGTAATCCTCCCAAATCA | CTTTATCCCGGCGTTTCATC |
| **ACTA2** | GATCTGGCACCACTCTTTCTAC | CAGGCAACTCGTAACTCTTCTC |
| **FN1** | TATGAGCAGGACCAGAAATAC | CCACTTCATGTTGTCTCTTC |
| **COL1A1** | GCTATGATGAGAAATCAACCG | TCATCTCCATTCTTTCCAGG |
| **COL1A2** | GTGGTTACTACTGGATTGAC | CTGCCAGCATTGATAGTTTC |
| **COL6A1** | AGCTCAATGTCATTTCTTGC | AGGTGTAATCTGGACACTTC |
| **COL6A2** | TACGGAGAGTGCTACAAG | TTCTCTCCTTTGAAGCCAG |
| **COL6A3** | AGCATCAAAGATAATGCCC | GAAAGTGTCTTGGTTGACTC |
| **TEADseqA ChIP** | GTTTGTTGCATGTGGTCCCT | GGACCTGGCACCGACTAAG |
| **TEADseqB ChIP** | TACTTCTTGGAGAGGGAGCG | AACCCACGGCCCCTATAAAA |
