# Supplementary material for: Proteomic profiling identifies a stromal TGF-β1/podoplanin axis as a driver of colorectal cancer progression
Source: J Exp Clin Cancer Res. 2025 Aug 22;44:247. doi: 10.1186/s13046-025-03496-3 (PMC12372361; doi:10.1186/s13046-025-03496-3)
Supplement: Supplementary file 2 — Supplementary Material 2 [file 13046_2025_3496_MOESM2_ESM.docx]

**Supplementary Table 2. Antibody list**

| **Name** | **Source** | **Identifier** |
| --- | --- | --- |
| **Rabbit polyclonal anti-pMLC2** | Cell Signalling Technology | Cat# 3671S, RRID: AB_330248 |
| **Mouse monoclonal anti-β-Actin** | Abcam | Cat# ab8226, RRID:AB_306371 |
| **Rat monoclonal anti-PDPN** | Thermo Fisher Scientific | Cat# MA5-16267, RRID:AB_2537784 |
| **Mouse monoclonal anti-α-SMA** | Sigma-Aldrich | Cat# A2547, RRID:AB_476701 |
| **Rabbit polyclonal anti-FN1** | Sigma-Aldrich | Cat# AB1954, RRID:AB_2105708 |
| **Rabbit monoclonal anti-COL1A1** | Cell Signalling Technology | Cat# 72026, RRID:AB_2904565 |
| **Mouse monoclonal anti-COL6A1** | Santa Cruz Biotechnology | Cat# sc-377143, RRID:AB_2783834 |
| **Mouse monoclonal anti-Vimentin** | Sigma-Aldrich | Cat# V6389, RRID:AB_609914 |
| **Mouse monoclonal anti-YAP/TAZ** | Santa Cruz Biotechnology | Cat# sc-101199, RRID:AB_1131430 |
| **Mouse monoclonal anti-RhoA-GTP** | NewEast Biosciences | Cat# 26904, RRID:AB_1961799 |
| **Rabbit monoclonal anti-YAP1** | Abcam | Cat# ab205270, RRID:AB_2813833 |
| **Mouse monoclonal anti-TAZ** | Abcam | Cat# ab224239, RRID:AB_2889852 |
| **Mouse monoclonal anti-Histone H3** | Abcam | Cat# ab4729, RRID:AB_2118291 |
