# Supplementary material for: Proteomic profiling identifies a stromal TGF-β1/podoplanin axis as a driver of colorectal cancer progression
Source: J Exp Clin Cancer Res. 2025 Aug 22;44:247. doi: 10.1186/s13046-025-03496-3 (PMC12372361; doi:10.1186/s13046-025-03496-3)
Supplement: Supplementary file 3 — Supplementary Material 3 [file 13046_2025_3496_MOESM3_ESM.docx]

**Supplemental Table 3. Characteristics of the patients**

| **COHORT 1** | | | |
| --- | --- | --- | --- |
| **Gender** | **Age** | **pTNM** | **Grade** |
| M | 88 | pT3N0M0 | G2 |
| M | 73 | pT2N0M0 | G2 |
| M | 82 | pT3N0M0 | G2 |
| M | 81 | pT3N0M0 | G2 |
| F | 80 | pT3N0M0 | G2 |
| F | 63 | pT3N0M0 | G2 |
| F | 64 | pT3N1aM0 | G2 |
| M | 64 | pT3N1aM0 | G2 |
| F | 61 | pT3N1aM0 | G2 |
| M | 74 | pT3N1AM0 | G2 |
| M | 67 | pT3N1cM0 | G1 |
| F | 79 | pT3PN0M0 | G2 |
| M | 40 | pT3PN1aMx | G2 |
| M | 83 | pT3PN1bMx | G2 |
| F | 85 | pT3PN2aMx | G2 |
| F | 79 | pT4APN1cMx | G2 |
| M | 76 | pT4APN1cMx | G2 |
| F | 77 | pT4APN2Mx | G2 |
| M | 74 | pT4bPN1bMx | G2 |
| F | 70 | pT4N2BM1A | G2 |

| **COHORT 2** | | | |
| --- | --- | --- | --- |
| **Gender** | **Age** | **pTNM** | **Grade** |
| M | 78 | pT1N0Mx | G1 |
| M | 81 | pT2N0Mx | G1 |
| M | 91 | pT2N0Mx | G2 |
| F | 81 | pT2N1Mx | G1 |
| M | 75 | PT2N0Mx | G1 |
| F | 71 | pT2N0Mx | G1 |
| F | 65 | PT3N1Mx | G2 |
| F | 87 | pT3N0Mx | G2 |
| F | 71 | pT3N1Mx | G2 |
| F | 65 | pT3N0Mx | G3 |
| M | 79 | pT3N1Mx | G2 |
| F | 87 | pT3N1Mx | G3 |
| F | 47 | pT3N1Mx | G2 |
| M | 93 | pT3N1Mx | G2 |
| M | 65 | pT3N0Mx | G1 |
| F | 75 | pT3N1Mx | G3 |
| F | 69 | pT3N2Mx | G3 |
| F | 91 | pT4aN1Mx | G2 |
| M | 61 | pT4aN1Mx | G3 |
| F | 68 | pT4bN1Mx | G2 |
