# Supplementary material for: Proteomic profiling identifies a stromal TGF-β1/podoplanin axis as a driver of colorectal cancer progression
Source: J Exp Clin Cancer Res. 2025 Aug 22;44:247. doi: 10.1186/s13046-025-03496-3 (PMC12372361; doi:10.1186/s13046-025-03496-3)
Supplement: Supplementary file 5 — Supplementary Material 5 [file 13046_2025_3496_MOESM5_ESM.pdf]

Supplementary Figure and Figure legends.

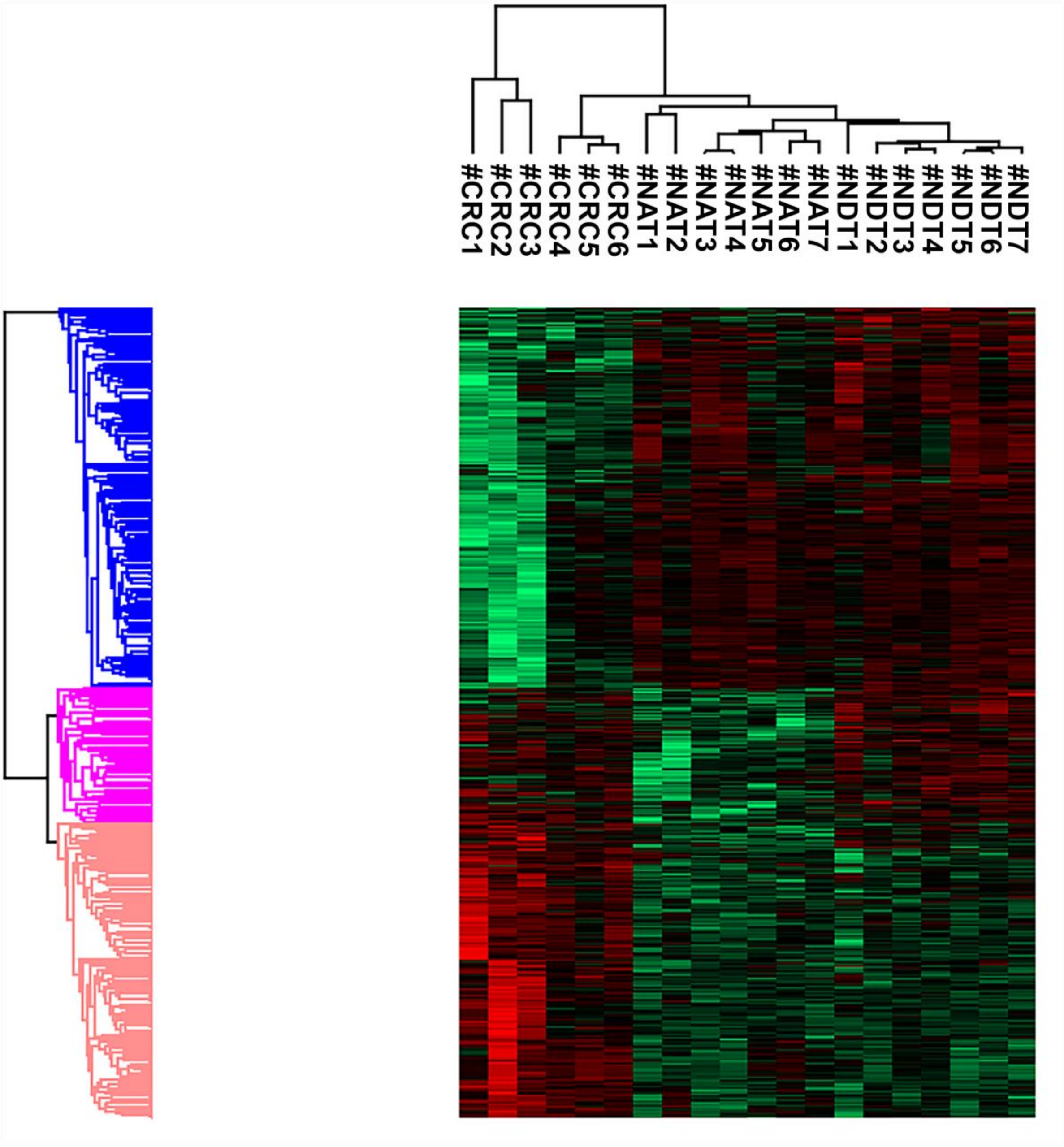

Supplementary Figure 1. Hierarchical clustering of protein identified by LC-MS/MS in CRC, normal tissue distant to the tumor and normal tissue adjacent to the tumor.

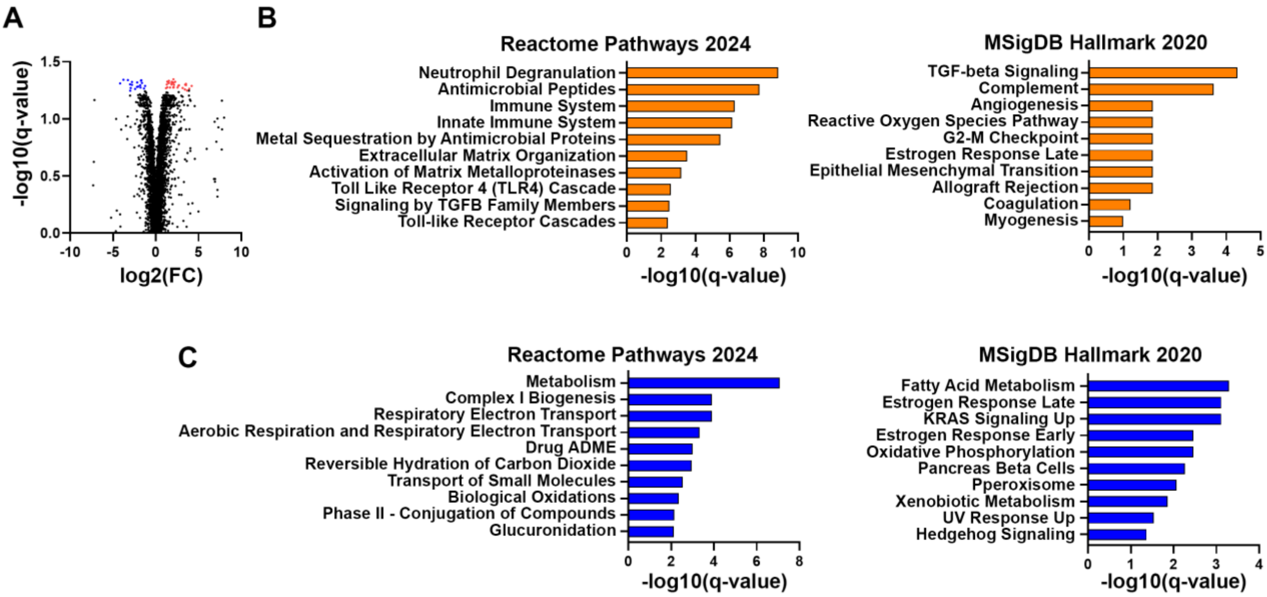

Supplementary Figure 2. A) Volcano plot displaying the log 2 fold change (x axis) against the −log 10 statistical q value (y axis) for all proteins differentially expressed between normal tissue adjacent to the tumor and CRC tissues. **B)** Enrichment pathway analysis of significantly up-regulated proteins in CRC versus NAT from the REACTOME 2024 and MSigDB Hallmark 2020 databases. **C)** Enrichment pathway analysis of significantly down-regulated proteins in CRC versus NDT from the REACTOME 2024 and MSigDB Hallmark 2020 databases.

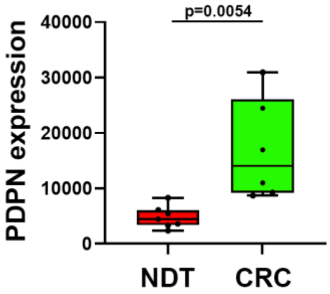

Supplementary Figure 3. PDPN protein expression in normal tissue distant to the tumor and CRC tissues. Data derive from the LC-MS/MS proteomic analysis.

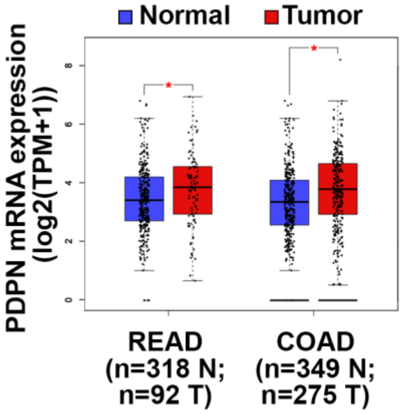

Supplementary Figure 4. PDPN gene expression in normal and tumor tissues from colon adenocarcinoma (COAD) and rectum adenocarcinoma (READ) patients and normal GTex colorectal tissues. Cancer data have been obtained from The Cancer Genome Atlas (TCGA Firehose Legacy). The analysis was performed by GEPIA2 with a p-value cutoff of 0.05.

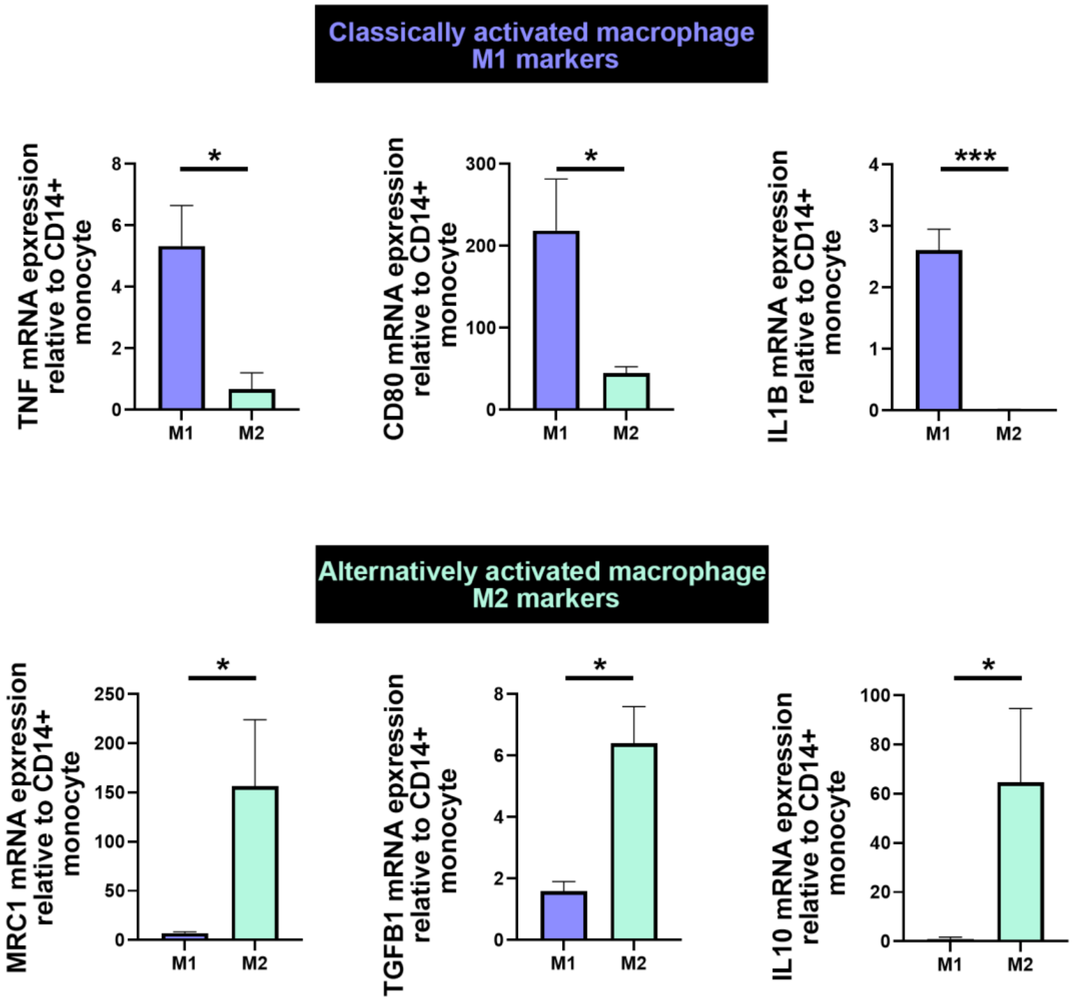

Supplementary Figure 5. RT-qPCR of markers of classically M1 (TNF, CD80, IL1B) and alternatively M2 (MRC1, TGFB1, IL10) activated macrophages derived from the differentiation protocol used in this study. Unpaired t test, n=3. * $p < 0.05$, *** $p < 0.001$.

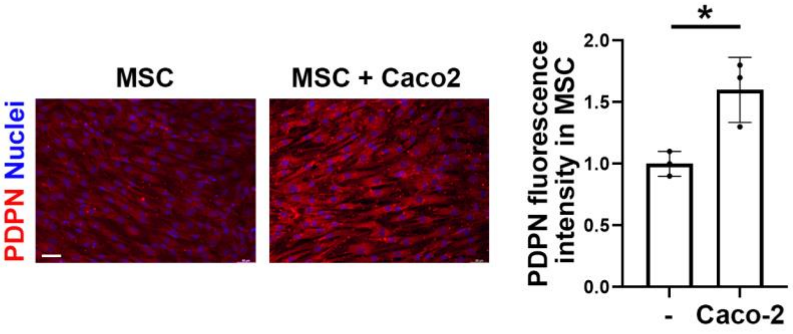

Supplementary Figure 6. Representative immunofluorescence staining of PDPN in MSC cultured alone or co-cultured with Caco-2 adenocarcinoma cells. Nuclei were counterstained with Hoechst. Scale bar is 50 µm. Quantification of PDPN staining intensity is shown. Unpaired t test, n=3.

Upstream Regulator	Expr FC	Activation z-score	Target Molecules in Dataset
TGFB1	4,821	2,156	ARG1,BCL3,CADM1,CALB2,CAMP,CD40,CLDN7,CNN2,CYBA,DCN
NOS2	3,231	2,361	LCN2,MMP9,NOS2,S100A8,SYP,TGFB1,TIMP1
STAT1	2,578	2,359	ACSL4,ARG1,BCL3,CD40,FCER1G,GBP5,ICAM1,ITGAX,KLF4,LCN2
lipopolysaccharide		4,646	ABCB1,APOA4,ARG1,AZU1,BCL3,BPI,CAMP,CD163L1,CD40,CD82
TNF		4,621	ACSL4,ARG1,AZU1,BCL3,CA12,CA2,CAMP,CD163L1,CD40,CD82
IL17A		3,774	BCL3,CAMP,CD40,GBP5,ICAM1,LCN2,LTBP2,LTF,MMP8,MMP9
IL1B		3,565	ARG1,BCL3,CA12,CD40,CD82,CYBA,DCN,ENG,GBP5,HSD17B2
OSM		3,542	ARG1,BCL3,CADM4,CALB2,CAMP,COX7B,FMO5,GAP43,HSD11B2,ICAM1
IFNG		3,464	ARG1,BCL3,CALB2,CD163L1,CD40,CYBA,DCN,FCER1G,FCN1,GBP5
TGM2		3,371	CA2,DEFA3,FCER1G,ITGAM,ITGAX,MMP8,MMP9,OAS2,PARP9,RNF213

Supplementary Figure 7. Top 10 Upstream regulator of the differentially expressed proteins between normal tissue distant to the tumor and CRC. The analysis was performed by Ingenuity Pathway Analysis software.

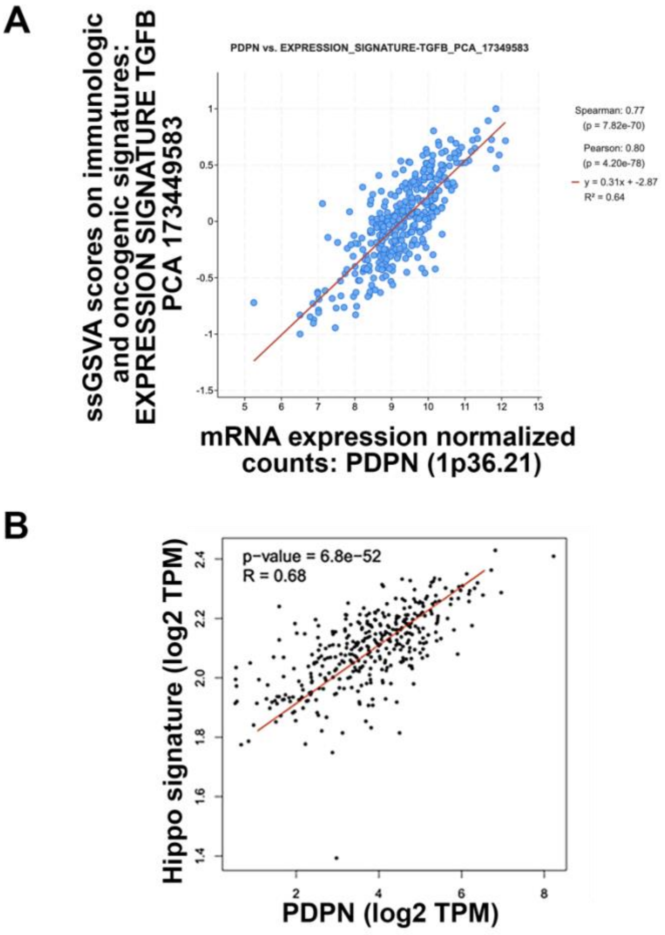

Supplementary Figure 8. A) Pearson correlation between PDPN mRNA expression and HIPPO signaling pathway signature in CRC tissues. Data derive from CRC TCGA Firehose Legacy. **B)** Pearson correlation between PDPN mRNA expression and TGF-β signaling pathway signature in CRC tissues. Data derive from single-sample Gene Set Enrichment Analysis (ssGSEA) analysis obtained by cBioportal (Colon Sidra-LUMC AC-ICAM dataset [1]).

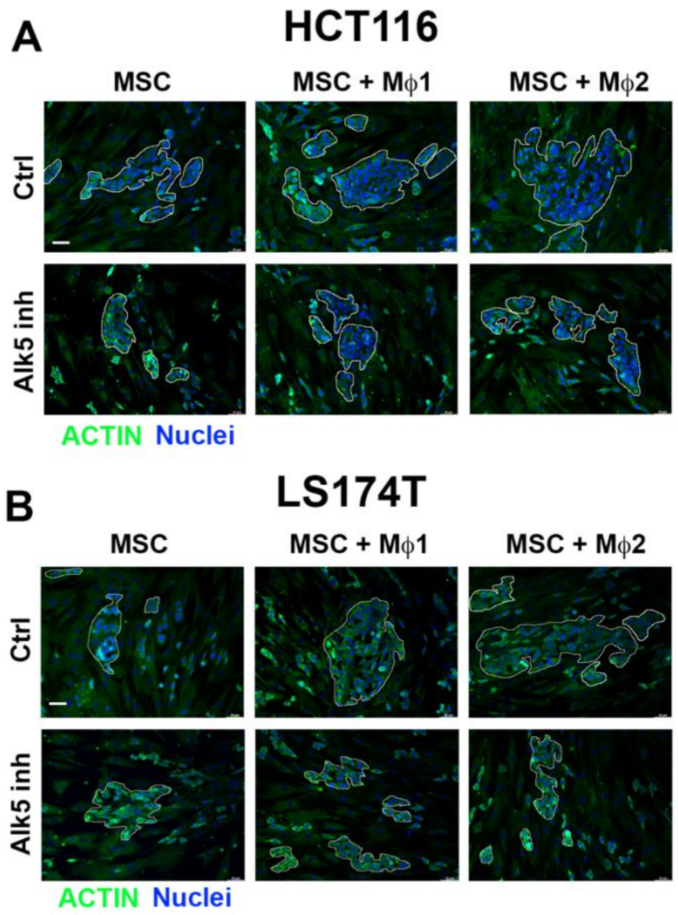

Supplementary Figure 9. Representative immunofluorescence staining of actin (green) and nuclei counterstaining (blue) of MSC/HCT116 co-cultures **(A)** and MSC/LS174T co-cultures **(B)**. Scale bar is 50 μm. Prior to colon adenocarcinoma cell seeding as monocellular suspension, MSC feeders were preconditioned or not with classically M1 or alternatively M2 activated macrophages. Where indicated MSC were pretreated with 10 μM TGF-β1 receptor ALK5 inhibitor.

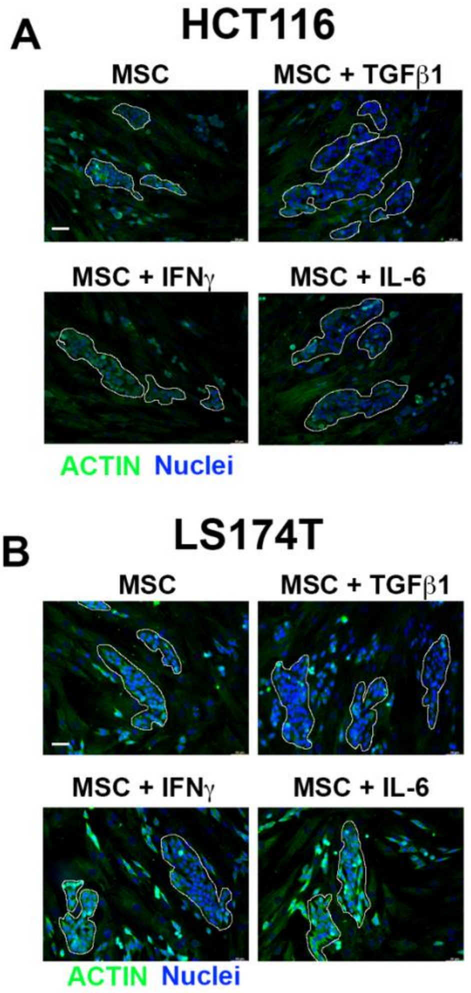

Supplementary Figure 10. Representative immunofluorescence staining of actin (green) and nuclei counterstaining (blue) of MSC/HCT116 co-cultures **(A)** and MSC/LS174T co-cultures **(B)**. Scale bar is 50 µm. Prior to colon adenocarcinoma cell seeding as monocellular suspension, MSC feeders were preconditioned or not with 10 ng/mL TGF-β1, 40 ng/mL IFNγ or 40 ng/mL IL-6.

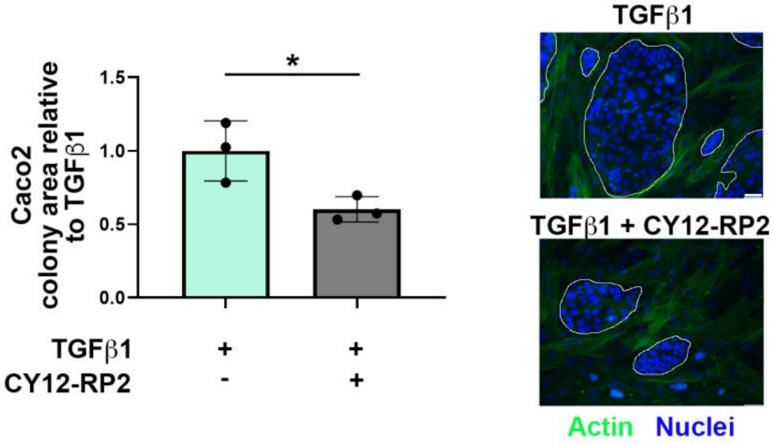

Supplementary Figure 11. Caco-2 cell colony area after 48 hours of direct co-cultures on MSC pre-conditioned with 10 ng/mL TGF-β1 in presence or not of 50 μM PDPN-antagonist peptide CY12-RP2. Unpaired t-test, n=3. The representative immunofluorescence staining of actin (green) and nuclei counterstaining (blue) of MSC/Caco-2 co-cultures are shown. Scale bar is 50 μm. * $p < 0.05$.

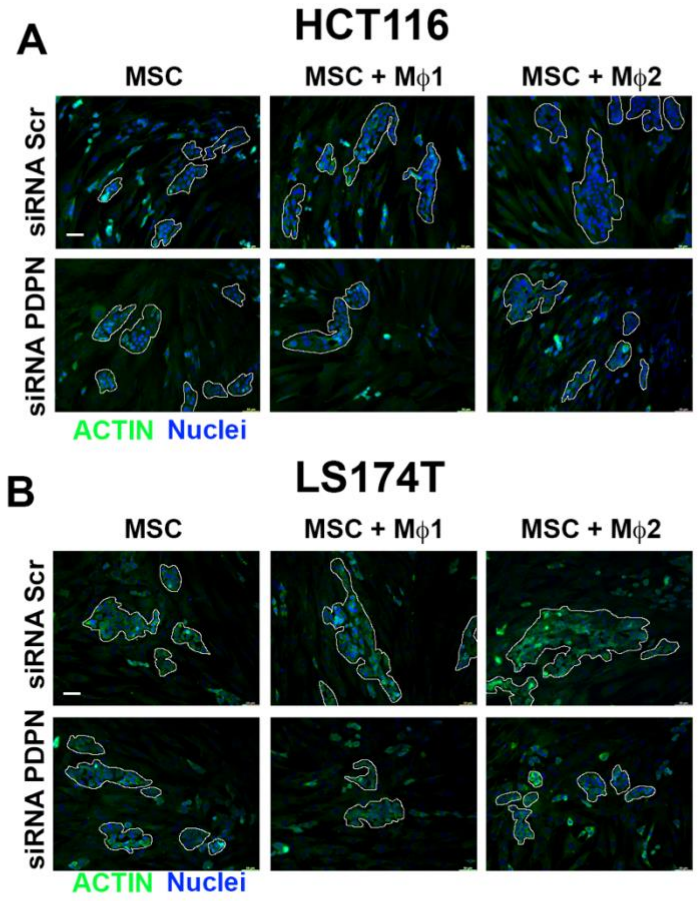

Supplementary Figure 12. Representative immunofluorescence staining of actin (green) and nuclei counterstaining (blue) of MSC/HCT116 co-cultures **(A)** and MSC/LS174T co-cultures **(B)**. Scale bar is 50 μm. Prior to colon adenocarcinoma cell seeding as monocellular suspension, MSC feeders were preconditioned or not with classically M1 or alternatively M2 activated macrophages. Where indicated MSC were pretreated with scamble siRNA or PDPN specific siRNA.

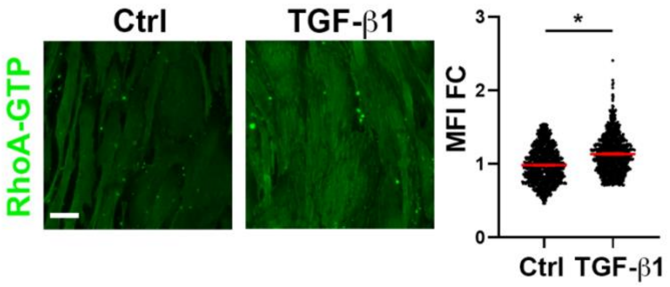

Supplementary Figure 13. Immunofluorescence analysis of active RhoA-GTP in MSC treated or not with 10 ng/mL TGF-β1. The quantification of RhoA-GTP staining intensity is shown. Data derive from 3 independent experiments and a minimum of 40 cells per experiment were analyzed. Unpaired t-test. Scale bar is 20 μm.

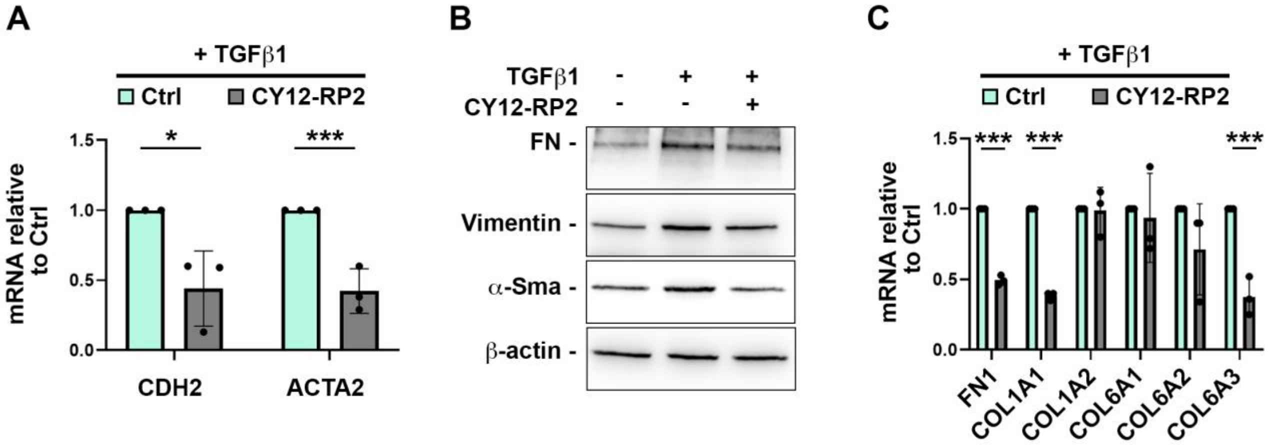

Supplementary Figure 14. A) RT-qPCR of EMT marker expression in MSC treated for 24 hours with 10 ng/mL TGF-β1 in presence or not of 50 μM PDPN-antagonist peptide CY12-RP2. Unpaired t test, n=3. **B)** Western blot of EMT markers fibronectin (FN), vimentin and α-Sma in MSC treated as in A. β-actin was used as loading control. **C)** RT-qPCR of ECM expression in MSC treated for 24 hours with 10 ng/mL TGF-β1 in presence or not of 50 μM PDPN-antagonist peptide CY12-RP2. Unpaired t test, n=3. * $p < 0.05$, *** $p < 0.001$.

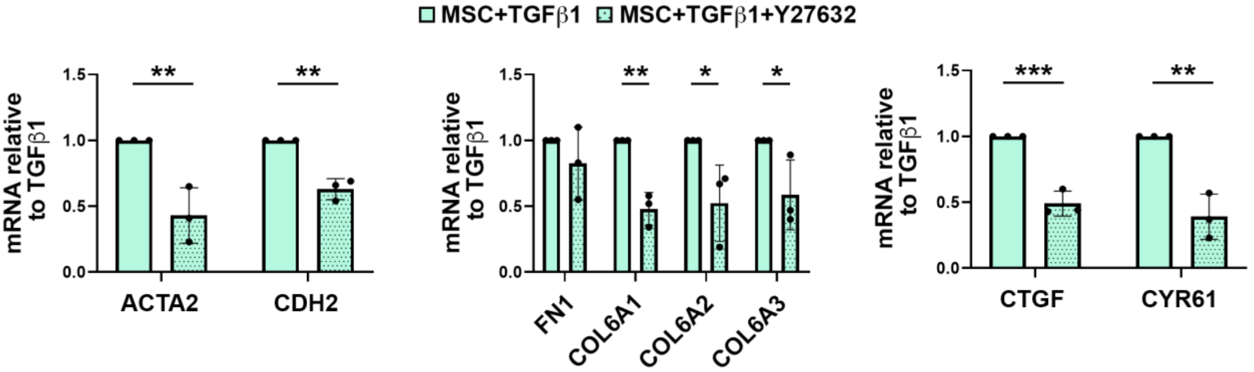

Supplementary Figure 15. RT-qPCR in MSC treated for 24 hours with 10 ng/mL TGF-β1 in presence or not of 10 μM ROCK inhibitor Y27632. Unpaired t test, n=3. * $p < 0.05$, *** $p < 0.001$.

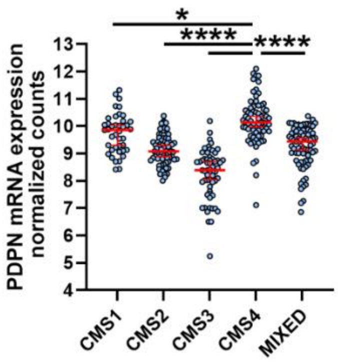

Supplementary Figure 16. PDPN mRNA expression in CRC tissues stratified on Consensus Molecular Subtypes (CMS). Data have been extracted from the Colon Sidra-LUMC AC-ICAM dataset [1]. * $p < 0.05$, **** $p < 0.0001$.

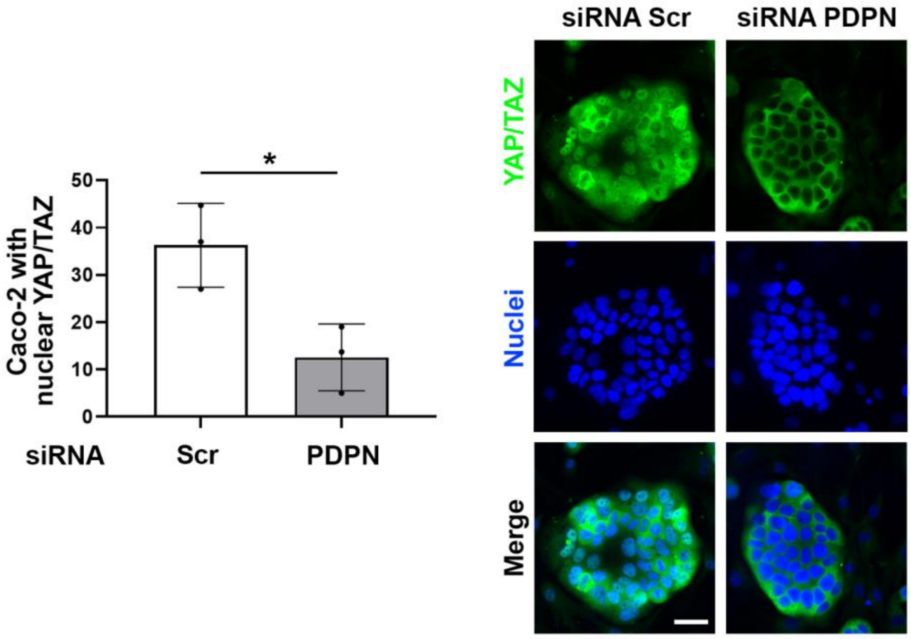

Supplementary Figure 17. Percentage of Caco-2 displaying a reduction of nuclear YAP/TAZ localization when grown on PDPN-silenced stromal feeders. Unpaired t test, n=3. * $p < 0.05$.

References

1. Roelands, J. *et al.* An integrated tumor, immune and microbiome atlas of colon cancer. *Nat Med* **29**, 1273-1286 (2023).
